# Fine-scale recovery of mite communities following local extinctions on grasslands

**DOI:** 10.1371/journal.pone.0357403

**Published:** 2026-09-01

**Authors:** Veronika Gergócs-Winkler, Norbert Flórián

**Affiliations:** HUN-REN Centre for Agricultural Research, Institute for Soil Sciences, Budapest, Fehérvári út, Hungary; Aarhus University, DENMARK

## Abstract

Agricultural management can substantially reduce soil biodiversity, potentially impairing key ecosystem processes. Understanding the capacity of soil biota to recover after severe disturbance is therefore essential for evaluating soil resilience. Soil-dwelling mite communities are known to respond sensitively to environmental change. We investigated the recovery of soil mite communities in a newly planted grassland using half-open field mesocosms. Grass mixtures were cultivated over three consecutive eight-month periods in chernozem and sandy soils. Within each system, mite assemblages in minimally disturbed soil were compared with those in experimentally defaunated soil, while a range of environmental variables was monitored. This design enabled the assessment of short-term local recovery potential. Mesostigmata (mean ± standard deviation of defaunation effect size, ES= + 0.07 ± 0.91) and Heterostigmata (Pygmephoroidea, ES= + 0.20 ± 0.61; Tarsonemidae, ES = −0.05 ± 0.36) recovered rapidly, likely due to efficient dispersal and high population growth rates. Their abundance was generally positively associated with vegetation biomass, suggesting improved microhabitat conditions and resource availability. In contrast, oribatid mites showed consistently slow and often incomplete recovery (ES = − 1.08 ± 0.66), highlighting the constraints imposed by limited dispersal ability and slow life cycles. Their recovery was further modulated by edaphic conditions, with more favourable outcomes in chernozem than in sandy soil. Overall, these results show that soil mite recovery is shaped by the interaction between taxon-specific life-history traits and environmental conditions. The findings further demonstrate that mite communities respond differently to disturbance, highlighting the need to account for functional differences among taxa when assessing recovery.

## Introduction

Soil is one of the most important natural resources for agricultural production; however, agricultural practices can also cause significant degradation of soil quality [1]. Tillage, high-input fertilisation, and herbicide application are widely used to meet the continually increasing demand for crop production [2,3]. Although effective in the short term, these practices can adversely affect the physical, chemical, and biological properties of soil, thereby undermining soil health and, ultimately, the sustainability of crop production [4]. One of the most critical consequences of intensive agricultural management is the loss of soil biodiversity [5]. Soil biota play essential roles in key ecological processes, including organic matter decomposition, nutrient cycling, and biological pest control [6,7]. The disruption or loss of members of the complex soil food web impairs these processes, reducing ecosystem functioning and making crop production systems less efficient and more dependent on external inputs [6]. Disturbances associated with agricultural management are, to a large extent, unavoidable in agroecosystems. Therefore, maintaining long-term soil health requires a thorough understanding of the regenerative capacity of soil biota following such disturbances. In particular, it is essential to determine both the mechanisms and the rate of recovery of soil communities in order to assess the severity and persistence of management-induced impacts. In the context of soil health monitoring, it is also crucial to understand the time required for soil animal communities to re-establish following disturbance, as this determines the resilience of soil ecosystem functioning [8].

Soil-dwelling mites are among the most abundant arthropods in agricultural soils [9] and are considered important indicators of soil biological recovery [10]. To investigate the regenerative capacity of soil biota in abandoned cropland, we focused on soil-dwelling mites. They constitute a highly diverse group of microarthropods, exhibiting a wide range of ecological tolerances, resource requirements, body sizes, life-history strategies, and functional roles [11,12]. The majority of soil-dwelling mites belong to three principal orders: Mesostigmata, Trombidiformes (with the largest group of Prostigmata), and Sarcoptiformes (containing Endeostigmata and Oribatida) [11]. Soil mites play significant roles in soil ecological processes. Many species feed on organic matter, thereby contributing to decomposition (e.g., Prostigmata, [12,13]). Others consume soil bacteria and fungi, influencing the activity and structure of soil microbial communities (e.g., Oribatida, [14]). In addition, predatory mites, particularly within the Mesostigmata, contribute to the regulation of soil-dwelling pest populations [15].

The sensitivity of these mite groups to disturbance is very variable. Species within the suborder Prostigmata are often reported as effective colonisers following various disturbances, such as fire [16] and land recultivation after mining [17,18]. They may also reach high abundances in agricultural soils [19,20]. Mesostigmata are frequently considered even more sensitive to disturbance than some prostigmatid groups. Reduced densities have been observed after several years of cultivation on post-industrial dumps [18] and spoil heaps [21], and their abundance is often higher in grasslands than in adjacent croplands [22]. However, some studies have reported no significant differences in mesostigmatid abundance between agricultural management types [23], indicating context-dependent responses. Oribatida are generally regarded as the most sensitive group. They exhibit slow recovery following forest disturbance [24–26], drought [27,28], and post-mining restoration [18]. Consistent with this sensitivity, oribatid mites often show low diversity and density in intensively managed agricultural fields [23]. Furthermore, life-history traits may influence their recolonisation capacity, with sexually reproducing species generally assumed to be more rapid colonisers than parthenogenetic species [29].

The regeneration processes of soil-dwelling mite communities after disturbances remain poorly understood at fine spatial scales. Existing research has often focused on describing soil microarthropod communities under different management regimes [23] or on assessing succession after agricultural abandonment, by comparing natural grasslands with abandoned fields of different ages [10,30,31]. However, these studies generally investigate relatively large spatial scales (10–1000 m) and largely overlook the local processes through which soil biota recover from agricultural disturbances. These disturbances may lead to local extinctions and thereby alter the spatial structure of mite communities. Recovery at this scale may occur via two pathways [32]. First, a portion of the soil-dwelling mites may survive disturbance in either adult or egg form [9] and subsequently resume reproduction. Second, locally extinct patches may be recolonised through active or passive dispersal from surrounding areas [33].

The pronounced spatial patchiness of soil mites observed in arable fields [34] likely reflects a dynamic balance between local extinction and recolonisation shaped by disturbance frequency and recovery time [35]. Yet similar patchiness is also common in undisturbed habitats. Even in the absence of disturbance, soil microarthropods often exhibit strong spatial autocorrelation at finer spatial scales (5–20 m) [34,36], where environmental variables appear to exert a weaker influence on pattern formation [37]. This suggest that stochastic dispersal and other neutral processes contribute substantially to the formation and maintenance of patchy distributions.

Understanding dispersal is therefore essential for explaining both the recovery of locally extinct patches and the spatial organisation of soil mite communities. Furthermore, local colonisation processes may provide important insights into the mechanisms underlying broader-scale spatial patterns [38]. Nevertheless, our understanding of the dispersal abilities of soil mites remains limited [36] as only a low number of studies have examined the local dispersal and short-term recovery capacity of soil mites after disturbance [39,40]. Clarifying the role of dispersal in community reestablishment is therefore crucial for understanding the resilience of soil mite communities in disturbed areas. Here, resilience is defined according to the concept of engineering resilience [8], and is defined as the rate at which soil-dwelling mite groups return to their pre-disturbance abundance following disturbance [32].

The recolonisation dynamics of soil mites, following disturbance, can reveal the resilience of soil communities and the factors that shape community assembly. In this study, we investigated how soil mite communities recover following experimental disturbance in a grassland system established on former agricultural land, over three consecutive periods, each lasting eight months. We tested three hypotheses. First, we hypothesised that most major mite groups would recolonise disturbed soil relatively quickly, approaching the community structure observed in undisturbed soil, as reported in similar studies [41]. However, as a second hypothesis we predicted that recovery trajectories would differ among mite groups, reflecting variation in life-history traits and dispersal abilities [12]. Third, we hypothesised that, at this spatial scale, recolonisation would be driven primarily by stochastic dispersal processes rather than by environmental variation among years and seasons [37].

## Materials and methods

### The mesocosms and the treatments

The study took place in two locations with two different soil types in Hungary: Őrbottyán (47°40′10.15″N, 19°15′12.15″E), calcareous sandy soil [Hungarian classification, WRB: Mollic Umbrisol (Arenic)]; Nagyhörcsök (46°51′59.69″N, 18°31′08.41″E), calcareous chernozem soil (WRB: Calcaric Phaeozem, [42]). Both locations were in a recently (2020) planted, small meadow (10 × 30 m) within an agricultural area (S1 Fig), which was one year old at the time of the study (2021). Both study sites are managed by the HUN-REN Centre for Agricultural Research, and therefore no additional permits or authorisations were required for conducting the experiments.

This experiment was originally designed to compare soil nitrogen cycling in the presence and absence of soil-dwelling mesofauna, using half-open field mesocosms planted with grass [43]. The absence of mesofauna was achieved by using previously defaunated soil within selected mesocosms. However, under field conditions, the complete exclusion of soil fauna from these defaunated mesocosms could not be maintained throughout the experimental period. Consequently, in addition to the original objectives, these circumstances provided an opportunity to investigate the recolonisation and recovery of defaunated soil by soil mites during the course of the experiment. Nevertheless, several aspects of the original experimental design were not directly relevant to the aims of the present study.

In terms of the structure of the mesocosms, they consisted of a plastic cylinder (height: 30 cm, diameter: 40 cm, wall width: 1 cm) and a white, cylinder-shaped, translucent mesh stretched above it (height: 50 cm, S1 Fig). The cylinder was covered with another mesh at the bottom in order to separate the mesocosm from the surrounding soil and buried in the soil (depth: 15 cm). There were two types of mesocosms: the defaunated and the control ones. The main difference between the control and defaunated mesocosms was the soil in the plastic cylinder. The cylinder contained soil from the surrounding meadow (cleaned of roots) at a depth of 20 cm. For the control mesocosm, this soil was mixed, and then taken into the mesocosm. For the defaunated mesocosms, the same amount of soil was previously treated in three ways in the three different years of the experiment. In February 2021, the soil was taken into a freezer (−18°C) for 2 weeks, and after melting, the soil was taken into 16 defaunated mesocosms. In January-February 2022, the soil was frozen (−18°C) and melted (10°C) two times for two weeks before taking it into the 10 mesocosms in the field. In February 2023, the soil was dried at 105°C for 24 hours, then rewetted and taken into the 10 defaunated mesocosms in the field. In all cases, subsampling after treatment confirmed the absence of mesofauna, indicating that defaunation was initially effective.

To limit faunal movement, control and defaunated mesocosms were fitted with meshes of different pore size (300 µm and 34 µm, respectively). However, exposure to field conditions led to partial degradation of the mesh material and loosening of the seals between the mesh and the cylinder walls. Consequently, soil fauna were able to enter, and likely exit, both mesocosm types from the surrounding environment over time. This led to substantial bidirectional movement in both control and defaunated mesocosms, despite their structural differences. Although the experimental design was originally intended to ensure the exclusion of fauna from defaunated treatments, these conditions instead enabled the assessment of recolonisation dynamics under near-natural field conditions across repeated experimental runs. This interpretation is supported by the consistent observation that, across years, individuals from multiple mite groups colonised the defaunated mesocosms, in some cases reaching abundances exceeding those recorded in the control treatments (see in Results).

Each year in March, the soil in both control and defaunated mesocosms was homogenised to prepare the seedbed. Then, a perennial grass mixture was sown into each mesocosm [40% *Festuca rubra* L., 20% *F. heterophylla* Lam., 20% *F. arundinacea* Schreb.*,* and 20% *Lolium perenne* L.]. The systems were then left undisturbed to enable grass establishment. There were two sampling occasions after four and eight months (in July and October) to monitor the soil variables and the recovery of soil animals. The grass was irrigated once or twice per week, as required. Although termed “control”, these mesocosms did not contain undisturbed soil, as the soil in control mesocosms was mixed each year. This approach was intended to simulate ploughing and reseeding, thereby isolating the processes governing soil recolonisation.

### Biological analyses

The sampling campaign (animal, plant, and soil samples) was conducted every year in July and in October (after 4 and 8 months). For microarthropods, a 400 cm^3^ cylindrical soil corer (diameter and depth = 8 cm) was used to take one sample from each mesocosm. Soil fauna was extracted using Berlese-extractor over a period of one week and preserved in 70% ethyl alcohol. To measure microbial biomass, separate 50 cm^3^ soil sample was taken (only in 2021 and 2022). On each sampling date, the aboveground vegetation was clipped to a height of 5 cm and removed for a parallel investigation.

**Fig 1:**
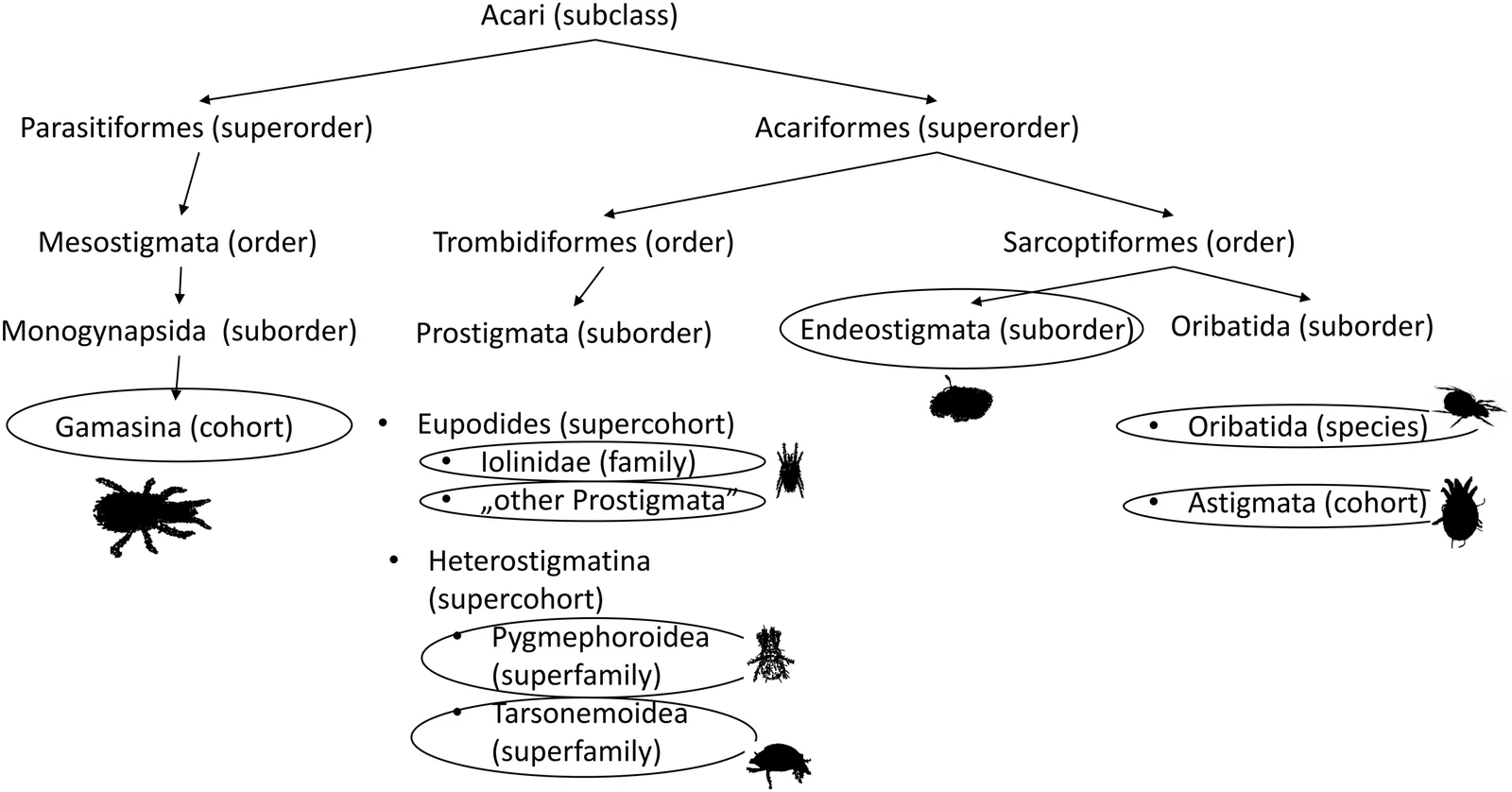
The identified mite groups. Soils at the study sites contained mites from four suborders. Based on their abundance and our taxonomic expertise, mites were identified at different taxonomic levels, as indicated by the circled groups. Gamasina and Astigmata were identified at the cohort level, Endeostigmata at the suborder level, and Oribatida at the species level. Prostigmata was divided into four groups because three of these groups occurred at particularly high densities during the experiment.

Mesofauna were investigated in the extracted samples under stereomicroscope (Delta Optical SZ-450-B) and mites were selected from other animals and soil particles and were enumerated. Mite groups were basically identified up to suborders based on Walter and Krantz [11], except for Iolinidae (Prostigmatina), Tarsonemidae (Heterostigmatina) families, Pygmephoroidea (Heterostigmatina) superfamily and Oribatida species. The high density of these families justified putting them in separate groups. In analyses about mite groups, we used the following groups: order Mesostigmata (only cohort Gamasina), Oribatida suborder, cohort Astigmata, other suborder Prostigmata (without Iolinidae and mainly Eupodidae and Tydeoidae), suborder Endeostigmata (mainly Nanorchestidae), and finally family Iolinidae, family Tarsonemidae and superfamily Pygmephoroidea (Fig 1). In addition, suborder Oribatida was identified at species level [44]. During the study, some specimens were treated with lactic acid and investigated with light microscope (Nikon Eclipse Ts2R). Reproduction mode and trophic guild of oribatid mites were determined by literature sources (e.g., [45,46]).

Microbial biomass was estimated by substrate-induced respiration (SIR) [43,47–49].

### Chemical analyses

Soil moisture was measured with Campbell Scientific device (HydroSense II Handheld Soil Moisture Sensor) in V/V%. For analysing soil chemical parameters (humus %, total N content, soil NO_3_^-^-N, soil NH_4_^+^-N) another 200 cm^3^ soil samples were taken from each mesocosm.

For soil chemistry analyses, soil total N content, humus %, soil ammonium (NH_4_^+^-N) and nitrate (NO_3_^—^N) contents were determined (see in [7]). For grass plant parameters, total plant biomass per mesocosm and total nitrogen content of plant tissue was measured. Steam distillation methods [50,51] were used to determine soil ammonium and nitrate contents and the total nitrogen content of soil samples. Soil humus content (%) was determined using the Tyurin method [52]. As humus % and SIR values showed little variation within the soil types and years, these parameters were only measured in 2021 and 2022.

### Statistical analyses

The taxonomic resolution varies across mite groups in this study, as some taxa were identified to suborder, others to family, and oribatid mites to species level. This inconsistency precludes a unified community analysis at the species level, which represents a limitation of the present study. Consequently, analyses were conducted separately for higher-level mite groups and oribatid mite species, in order to minimise potential confusion arising from the mixed taxonomic resolution.

All statistical analyses were performed in R Studio (R version 4.5.1) [53]. Mite group density (individuals per sample = individuals per 50 cm^2^) data were standardised separately for each group using Z-score transformation to account for differences in absolute density scales. Treatment (control vs defaunation) effects were calculated for each group within each year × month combination as the difference between mean standardized density in defaunated and control plots. Standard errors (SE) were computed assuming independent samples, and 95% confidence intervals were estimated as mean differences ± 1.96 × SE. Results were visualised using faceted plots to illustrate temporal and group-specific treatment effects using ggplot function from the ggplot2 package [54].

Environmental parameters, mite groups, and the dominant Oribatida species were analysed separately for each soil type and year. Effects of treatment (control vs defaunation) and month (after 4 months vs after 8 months) were analysed using linear models (lm) or, where assumptions were violated, generalised least squares models (gls) were used (nlme package, [55]). Model selection was based on comparisons using the anova() function. For gls models, a varIdent variance structure was applied to account for heteroscedasticity among years. Pairwise contrasts among years were calculated using the emmeans() functions from the emmeans package [56]. Mite group densities were visualised using barplots, while mean densities of oribatid species were presented as bar plots across soil types and years within each treatment × month combination, using ggplot2.

Relationships between mite groups and environmental variables were analysed using redundancy analysis (RDA) implemented with the rda() function in the vegan package [57]. Mite densities (individuals per sample) were Hellinger-transformed prior to analysis. Marginal effects were tested using the anova() function. To assess the influence of continuous environmental variables independently of categorical factors, separate RDAs were performed for each year and for the pooled dataset across all years; for 2021 and 2022, SIR and humus content were additionally included as explanatory variables. Relationships between individual mite groups and environmental variables were further explored using Spearman rank correlations [58].

Structural equation modelling (SEM) was performed using the piecewiseSEM package [59] to investigate direct and indirect pathways by which experimental factors and environmental variables influenced mite groups separately within each soil type. Selection of environmental predictors for inclusion in the SEMs was based on the results of lm, gls, and Spearman correlation analyses. Two separate SEMs were constructed: one for chernozem soil and one for sandy soil. Each model included the three categorical factors (treatment, year, and month) together with continuous predictor variables that showed strong associations with the respective response variables. The component linear models underlying the SEMs are listed in S1 Table. All continuous variables were standardised prior to analysis using the scale() function to ensure comparability of effect sizes and to obtain standardised path coefficients from the psem() function. Model fit was evaluated using the Chi-square test and Fisher’s C statistic, with p > 0.05 indicating adequate fit. The proportion of variance explained (R²) were calculated and are reported for each response variable. It should be noted that the number of replicates was unbalanced across years (n = 16 in 2021; n = 10 in 2022 and 2023), resulting in a total of N = 144 observations per soil type [2 treatments × 2 sampling time points × 16 replicates in 2021, plus 2 years × 2 treatments × 2 sampling time points × 10 replicates in 2022–2023]. Given this moderate sample size, particular attention was paid to model fit indices, the stability of parameter estimates, and R^2^ values as indicators of model reliability, rather than relying solely on conventional sample size thresholds.

## Results

### Recovery trajectories after defaunation

#### Mite groups (without Oribatida).

**Fig 2:**
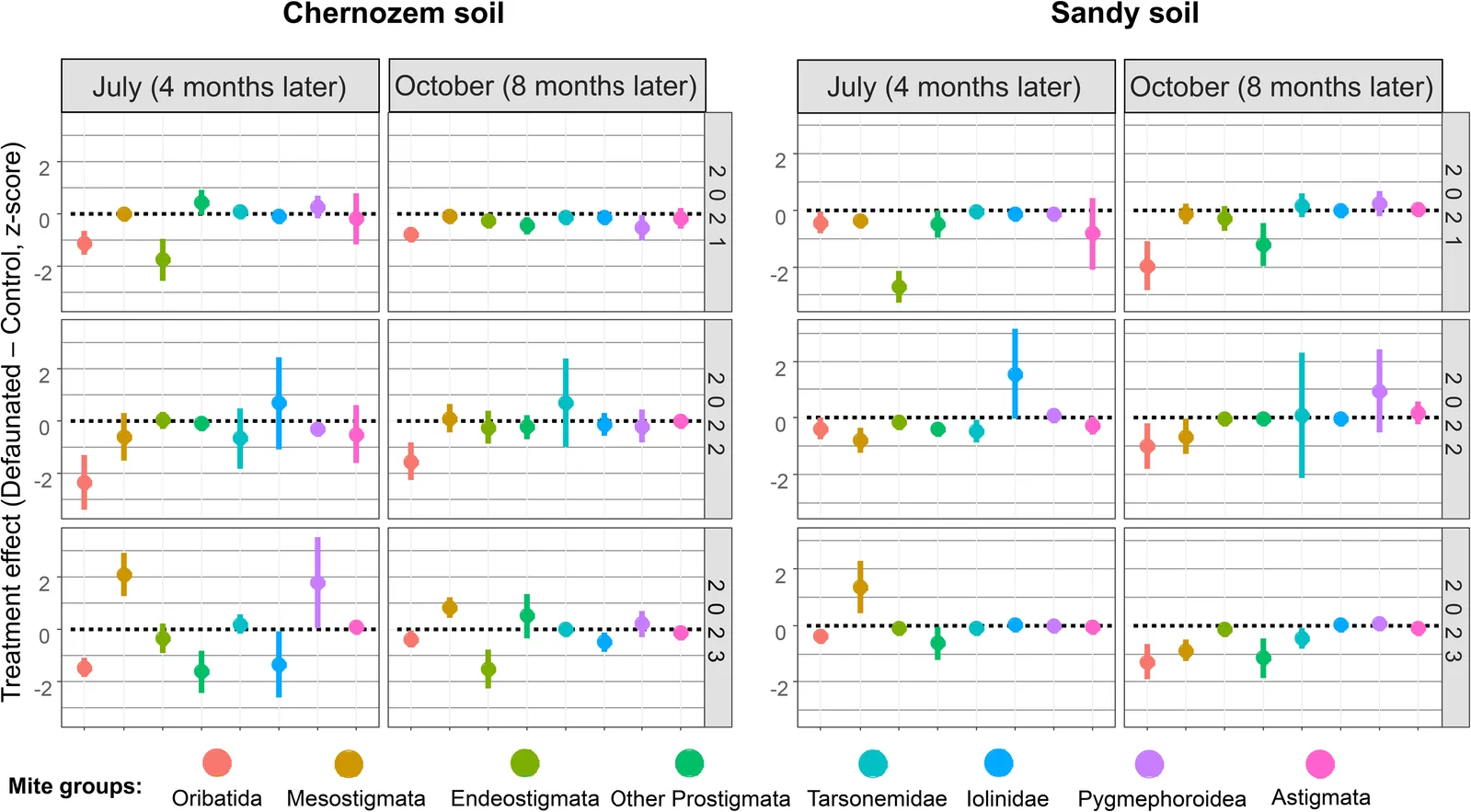
Treatment effects of defaunation on the density of mite groups across years and months in chernozem and sandy soils. Densities were standardised within each group using a Z-score transformation. Treatment effects were calculated as the mean difference between defaunated and control samples (defaunated − control) for each year × month combination. Points represent mean standardised differences, and error bars indicate 95% confidence intervals (± 1.96 × SE).

Mesostigmata, Endeostigmata and the group “other Prostigmata” displayed distinct patterns according to the colonisation and recovery abilities. Mesostigmatid mites were among the most abundant mite groups, with mean densities (±SD) of 46.8 ± 48.1 individuals per sample in sandy soil and 22.8 ± 28.4 individuals per sample in chernozem soil. Freezing defaunation (applied in 2021 and 2022) had only minor long-term effects on Mesostigmata, as indicated by the small effect sizes (ES) recorded eight months after defaunation (sandy soil: ES = −1.28 to 0.25; chernozem soil: ES = −0.41 to 0.66; Fig 2). Consistent with these results, Mesostigmata densities did not differ significantly between faunated and defaunated mesocosms during either year (S2 Fig). In contrast, drying defaunation (applied in 2023) resulted in more pronounced responses. In July 2023, Mesostigmata density was markedly higher in defaunated mesocosms than in control mesocosms in both soil types (sandy soil: ES= + 1.36 ± 0.93; chernozem soil: ES= + 2.08 ± 0.84, Fig 2); subsequently, trends diverged between soils. In chernozem soil, densities remained slightly higher in defaunated mesocosms (ES= + 0.12 ± 0.53) whereas in sandy soil they became lower than in faunated mesocosms (ES = −0.88 ± 0.37, S2 Fig, S2 Table).

Endeostigmata generally occurred at low densities throughout the study period, with mean densities of 9.5 ± 18.3 individuals per sample in sandy soil and 8.0 ± 12.2 individuals per sample in chernozem soil. An exception was observed in July 2021, when densities were markedly higher in control mesocosms (54.3 ± 19.5 and 28.2 ± 18.9 individuals per sample in sandy and chernozem soils, respectively) than in defaunated mesocosms, resulting in large negative effect sizes in both soil types (sandy soil ES = −2.71 ± 0.56; chernozem soil: ES = −2.55 ± 0.80; Fig 2, S2 Fig, S2 Table). A similar pattern was recorded in October 2023 in chernozem soil, where control mesocosms again supported higher Endeostigmata densities than defaunated mesocosms (S2 Fig). In contrast, other Prostigmata typically exhibited comparable densities in defaunated and control mesocosms, although occasional reductions were observed in defaunated mesocosm most notably in July 2023 (sandy soil: ES = −0.36 ± 0.59; chernozem soil: ES = −2.45 ± 0.81; Fig 2).

In contrast, four mite groups (Iolinidae, Pygmephoridae, Tarsonemidae and Astigmata) showed no significant differences between defaunated and control mesocosms in most cases (Fig 2; S2 Fig). The minimal differences between treatments persisted even when population densities were particularly high in both treatments. For example, in October 2022, Pygmephoroidea and Tarsonemidae reached exceptionally high densities in sandy soil, but their densities remained similar between control and defaunated mesocosms (Pygmephoridae: 795 ± 520 vs. 1222 ± 885 individuals per sample; Tarsonemidae: 113 ± 191 vs. 122 ± 201 individuals per sample, respectively). Likewise, in chernozem soil, densities of both groups were comparable between treatments (Pygmephoridae: 25.2 ± 40.0 vs. 15.7 ± 29.3; Tarsonemidae: 77.6 ± 47.1 vs. 129 ± 192 individuals per sample in control and defaunated mesocosms, respectively; S2 Fig). Similarly, Iolinidae exhibited very high densities in both treatments in sandy soil in July 2022: yet abundances did not differ significantly between treatments (362 ± 563 vs 768 ± 322 individuals per sample in control and defaunated mesocosms, respectively S2 Fig). Astigmata, in particular, occurred at very low densities in all plots with mean densities of only 0.7 ± 2.0 individuals per sample in sandy soil and 1.0 ± 2.0 in chernozem soil S2 Fig).

**Fig 3:**
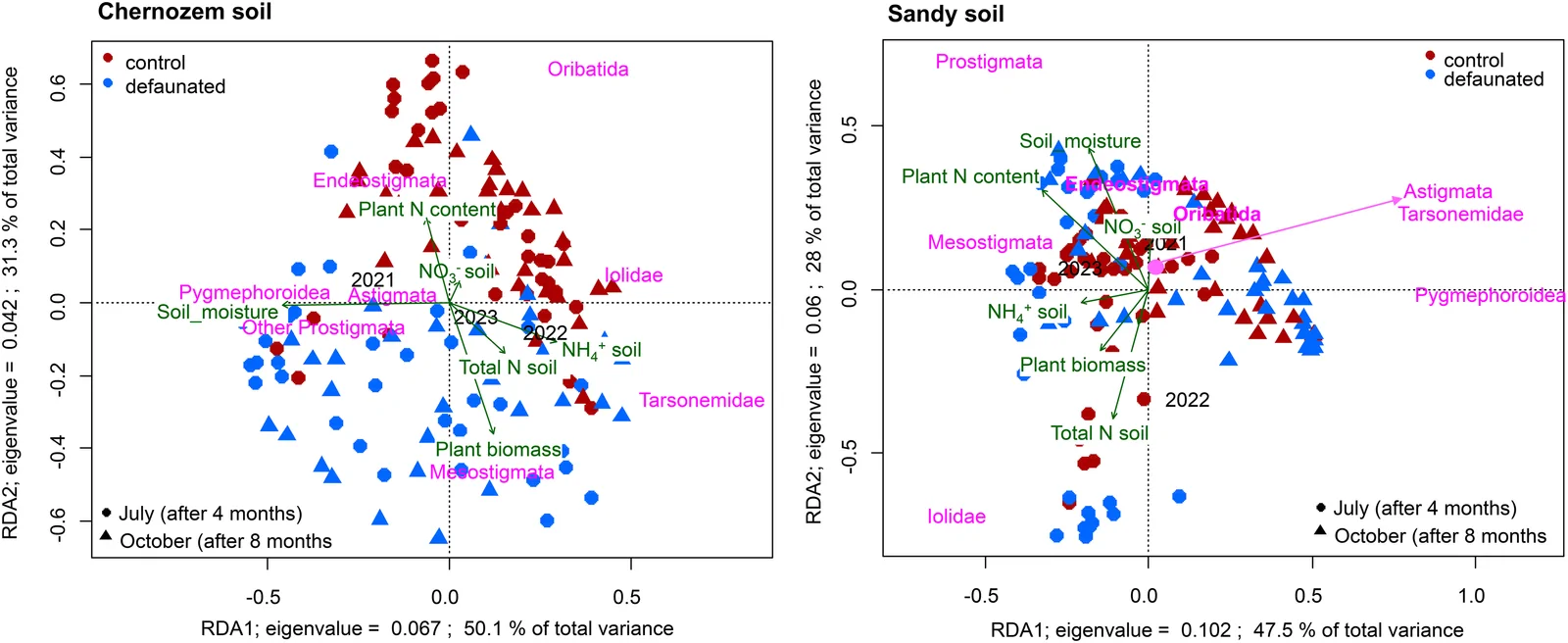
Redundancy analysis (RDA) of soil mite groups in relation to soil variables and the factors treatment, year and month. Additional details of the analyses are provided in S3 Table.

Redundancy analysis revealed differences in mite community composition between defaunated and control mesocosms in chernozem soil (Fig 3). These differences were primarily associated with the higher density of oribatid and endeostigmatid mites in control mesocosms and the higher densities of mesostigmatid mites in defaunated mesocosms. However, the separation between treatments varied among years. In sandy soil, time after disturbance (4 or 8 months after) was the main factor structuring community composition, with samples collected in October clearly separated from those collected in July. In contrast to chernozem soil, the separation between defaunated and control mesocosms in sandy soil was less pronounced (Fig 3, S3 Table).

#### Oribatid mites.

Oribatid mites proved to be the slowest recolonising mite group in our experiment (Fig 2). The mean densities of oribatid mites were consistently lower in defaunated mesocosms than in control mesocosms averaging 2.2 ± 8.7 versus 50.9 ± 62.9 individuals per sample in sandy soil and 8.3 ± 11.0 versus 50.6 ± 37.9 individuals per sample in chernozem soil, respectively. However, recovery dynamics differed between the two soil types. In chernozem soil, the difference in densities between defaunated and control mesocosms was greater four months after defaunation (ES = −0.67 to −3.39) than after eight months (ES = −0.07 to −2.26, Fig 2). In contrast, the opposite pattern was observed in sandy soil, where differences between treatments were smaller after four months (ES = 0.00 to −0.82) but became more pronounced after eight months (ES = −0.18 to −2.81). This pattern was primarily driven by seasonal changes in oribatid mite densities in control mesocosms. In chernozem soil, densities declined from July to October (59.5 ± 42.6 to 42.0 ± 30.8) whereas in sandy soil they increased markedly over the same period (20.0 ± 30.0 to 80.9 ± 71.9). In contrast, densities in defaunated mesocosms either increased or remained relatively stable. Nevertheless, density values showed considerable variability, with relatively high standard deviations also observed in the control plots (S2 Fig).

**Fig 4:**
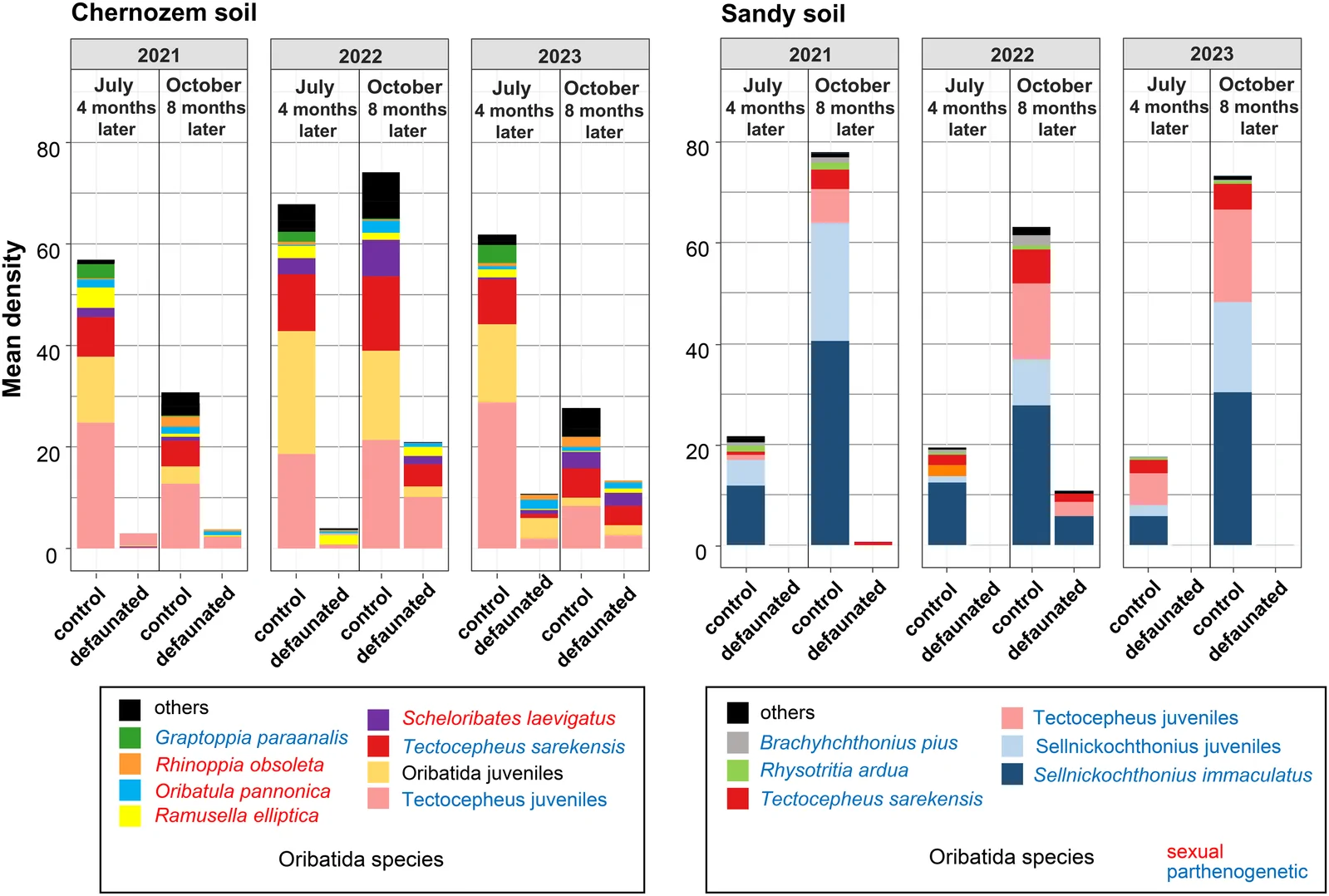
Mean density of dominant oribatid mite species in two soil types across three years and two months. Species names in blue indicate parthenogenetic taxa, whereas red names denote sexually reproducing species.

Chernozem soil supported higher oribatid diversity than sandy soil with 15 and 7 recorded species, respectively (Fig 4, S4 Table). In chernozem soil, adults and juveniles of *Tectocepheus velatus sarekensis* Trägårdh, 1910 constituted the dominant group (Fig 4). In the control mesocosms, juvenile oribatid mites consistently accounted for at least 50% of total densities, whereas in defaunated mesocosms this proportion tended to be lower (S3 Fig).

In contrast, sandy soil was characterised by the predominance of *Sellnickochthonius immaculatus* (Forsslund, 1942), while *T. v. sarekensis* was the second most abundant species (Fig 4). In control mesocosms, the proportion of juveniles was similar after four and eight months within each year, but increased from 2021 towards 2023. However, total densities remained consistently low in defaunated mesocosms (S3 Fig).

In chernozem soil, approximately half of the oribatid species were parthenogenetic (7 out of the 15 species, Fig 4, S5 Table), and the proportional density of parthenogenetic species in control mesocosms was also consistently close to 0.5 (S3 Fig). Colonising species in defaunated mesocosms included both sexually and asexually reproducing taxa, resulting in variable proportions of parthenogenetic species (S3 Fig). In contrast, in sandy soil the assemblage was composed almost exclusively of parthenogenetic species, with *Adelphacarus sellnicki* Grandjean, 1952 as the sole sexually reproducing exception (S3 Fig). Across both soil types, the majority of oribatid mites were secondary decomposers, primarily feeding on fungi and other microorganisms, and only partly on plant material (S5 Table).

### Relationships with environmental variables

The two soil types differed markedly in their physicochemical properties. Sandy soil had lower total nitrogen (1.3 ± 0.3 g/kg) and carbon/humus content (15.9 ± 1.4 g/kg) than chernozem soil (2.2 ± 0.3 and 30.9 ± 1.5 g/kg, respectively; S6 Table). Among the study years, the year 2021 showed the lowest soil nitrogen content in both soil types (sandy soil: 1.2 vs. 1.6 and 1.3 g/kg in 2021, 2022, and 2023, respectively; chernozem soil: 2.1 vs. 2.2 and 2.3 g/kg, respectively). In contrast, 2022 generally exhibited the greatest above-ground plant biomass values, with mean values of 13.6 g in sandy soil compared with 10.8 and 9.7 g in 2021 and 2023, respectively, while in chernozem soil biomass reached 15.4 g in 2022 compared with 8.86 and 16.2 g in 2021 and 2023, respectively. The year 2023 was distinguished by the highest soil NH4 ⁺ -N concentrations in both soil types, reaching 3.09 mg/kg in sandy soil and 3.86 mg/kg in chernozem soil, compared with 1.46 and 2.38 mg/kg in sandy soil and 1.26 and 2.39 mg/kg in chernozem soil in 2021 and 2022, respectively (standard errors are provided inS6 Table).

Redundancy analysis (RDA) indicated a significant relationship between environmental variables and mite community composition (Fig 3, S3 Table). Soil moisture, above-ground plant biomass, total soil nitrogen, and soil NH4 ⁺ -N content were identified as the most important and significant predictors for mite groups in both soil types (ignoring the factors: treatment, year, month; S3 Table). In contrast, soil NO3 − -N, substrate-induced respiration (SIR), humus content, and plant nitrogen content were not significant in either soil type (S3 Table, Fig 3). Distinct patterns of correlation between specific environmental variables and mite groups were evident within each soil type.

**Fig 5:**
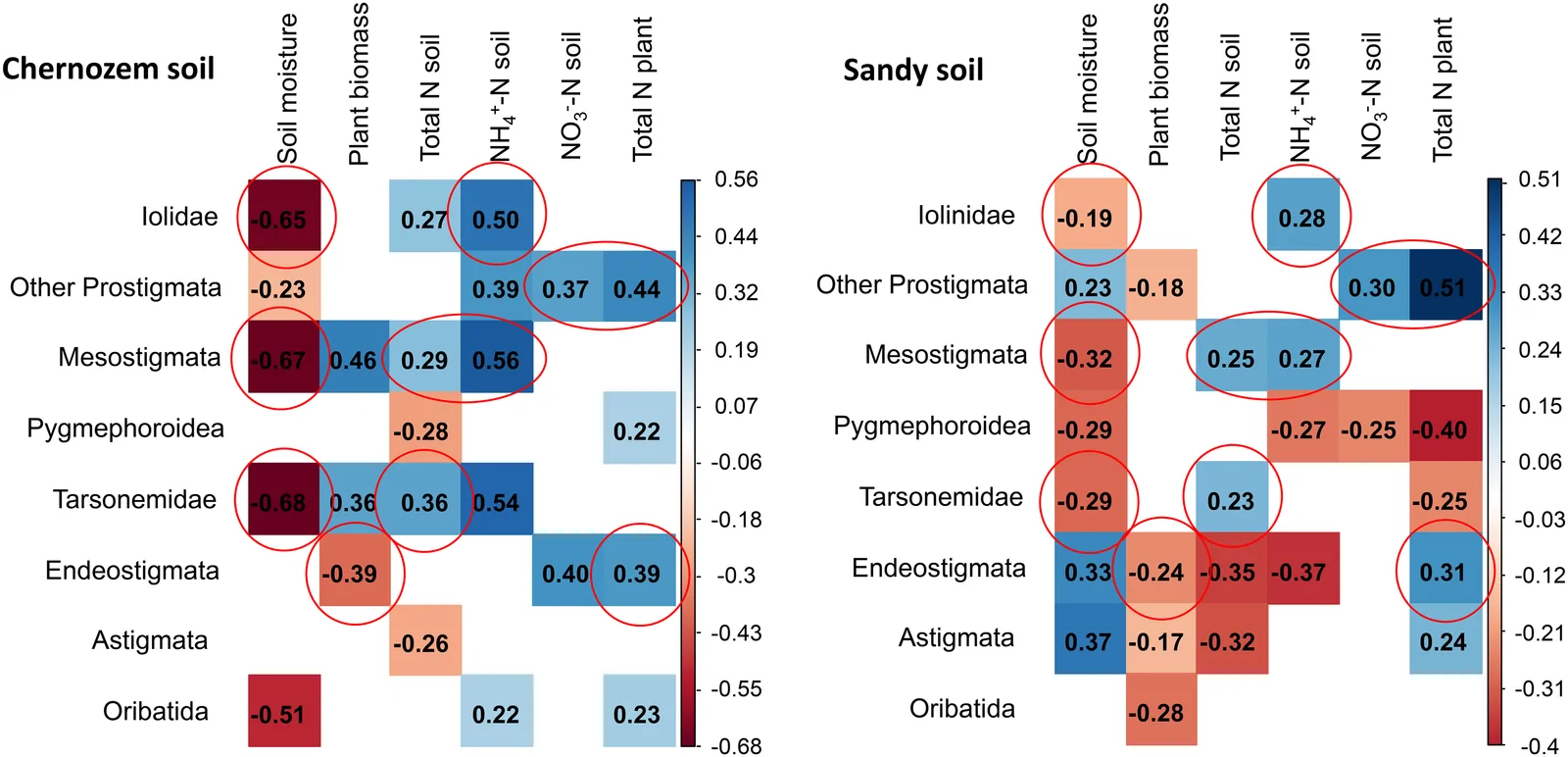
Spearman correlations between mite group densities (log(x + 1)) and the most important soil environmental variables across the two soil types. Only statistically significant (α < 0.05) correlations are shown; red circles indicate correlations that are shared between both soil types.

**Fig 6:**
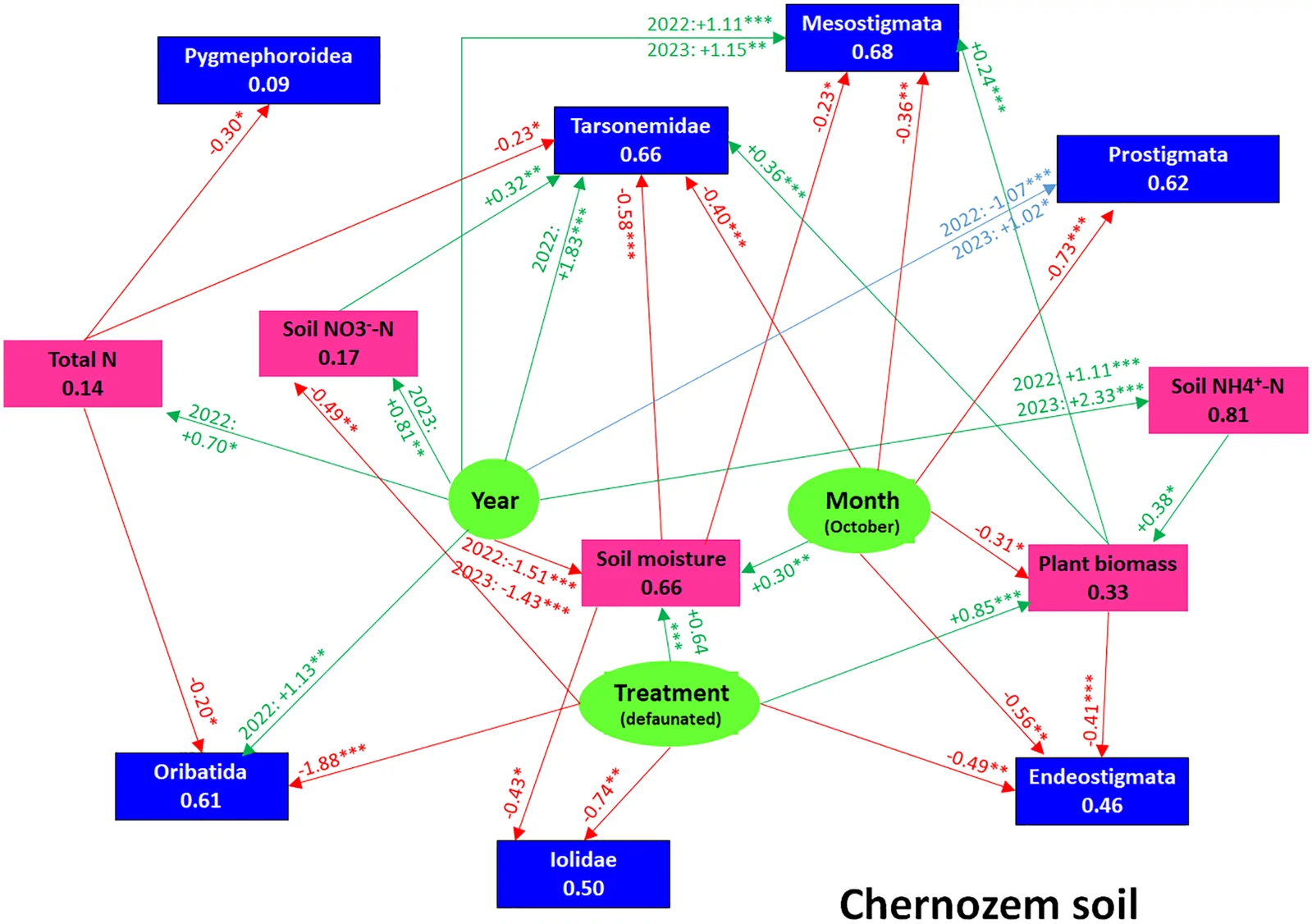
Structural equation modelling (SEM) illustrating significant linear relationships between mite groups and experimental factors (treatment, year, month), environmental variables in chernozem soil. Model fit: χ² = 34.33, p = 0.190, df = 28; Fisher’s C = 55.85, p = 0.405, df = 54. Arrows represent statistically significant effects. Positive effects are shown in green (+), negative effects in red (–) and factors with multiple levels and varying effects are indicated in blue. Significant parameters are shown with asterisks, *p < 0.05, **p < 0.01, ***p < 0.001. The proportion of variance explained (R²) is shown below the response variables. (Interactions between mite groups are ignored.).

**Fig 7:**
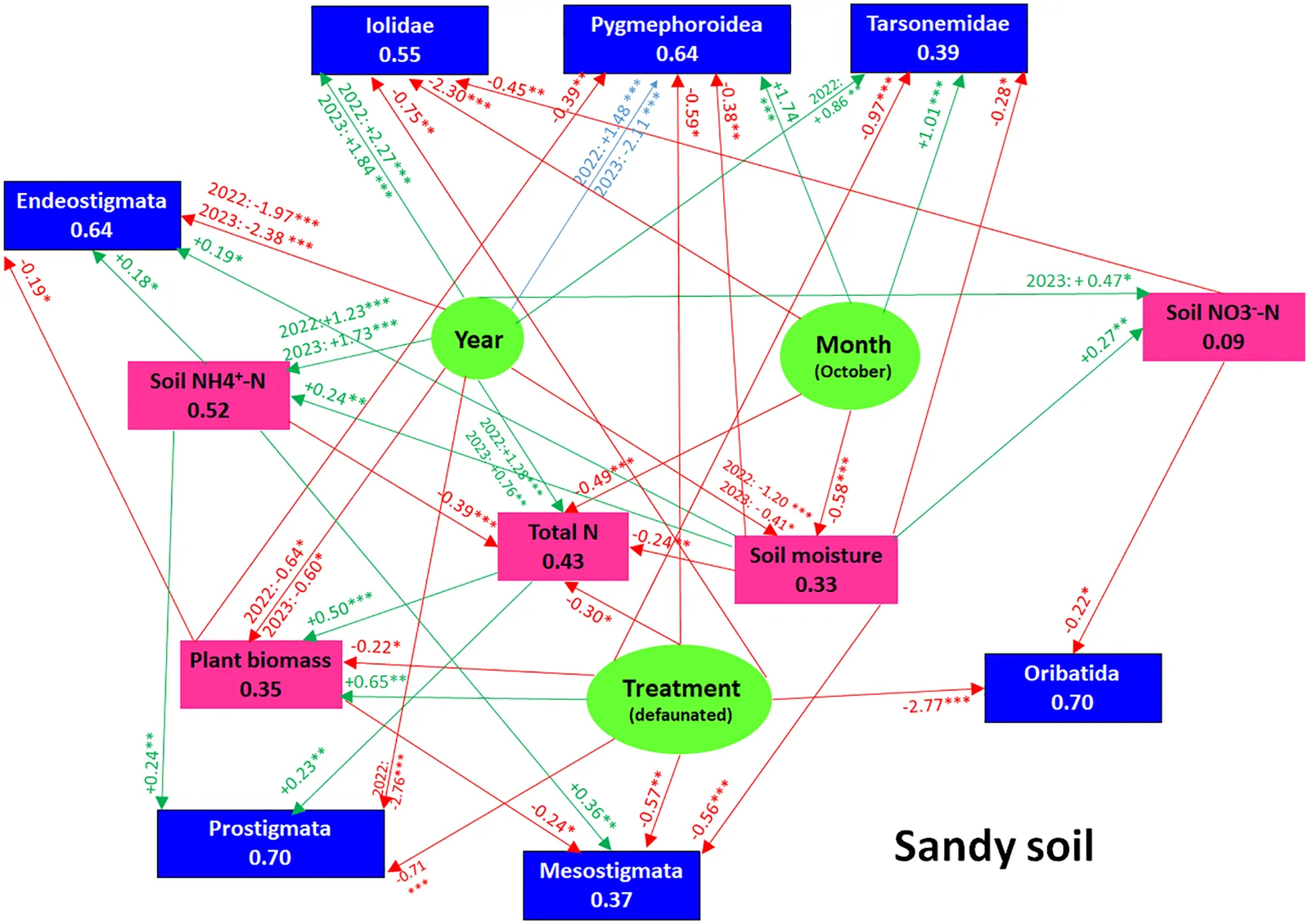
Structural equation modelling (SEM) illustrating significant linear relationships between mite groups and experimental factors (treatment, year, month), environmental variables in sandy soil. Model fit: χ² = 40.49, p = 0.241, df = 35; Fisher’s C = 49.57, p = 0.908, df = 64. Arrows represent statistically significant effects. Positive effects are shown in green (+), negative effects in red (–) and factors with multiple levels and varying effects are indicated in blue. Significant parameters are shown with asterisks, *p < 0.05, **p < 0.01, ***p < 0.001. The R^2^ value is shown below the response variables. (Interactions between mite groups are ignored.).

The main response of Mesostigmata was that soil moisture exerted a negative influence on their density in both soil types (r(sandy soil)=−0.32, p = 0.0002; r(chernozem)=−0.67, p<<0.001); Fig 5–7). Pygmephoroidea exhibited negative associations with total soil nitrogen content in chernozem soil (r = −0.28, p = 0.0006) (Fig 5–7), and in sandy soil their densities were affected by treatment and seasonal variation, showing peak densities in autumn (S2 Fig; Figs 6,7). Tarsonemidae were negatively associated with soil moisture in both soil types (r(sandy soil)=−0.29, p = 0.0005; r(chernozem)=−0.68, p<<0.001), and positively with plant biomass in chernozem soil (r = 0.36, p < 0.001), with densities reaching their highest levels in October. Endeostigmata displayed negative correlations with grass biomass (r(sandy soil)=−0.24 p = 0.004; r(chernozem)=−0.39, p<<0.001) and positive correlations with soil moisture in sandy soil (r=+0.33, p = 0.0001), with the relationship to plant biomass remaining independent of other factors (Fig 5–7). Soil moisture and plant biomass were themselves negatively correlated (Fig 3).

The family Iolinidae showed positive correlations with soil NH4 ⁺ -N (r(sandy soil)=+0.28, p = 0.0009; r(chernozem)=0.50, p<<0.001) in both soil types and with total nitrogen content in chernozem soil (r = 0.27, p = 0.0009) (Fig 5). However, SEM analyses indicated that these relationships were largely confounded by the effect of year (Figs 6,7). This pattern was particularly evident in sandy soil in 2022, when both total nitrogen content and Iolinidae density reached high values. Other Prostigmata exhibited only one consistent pattern across soil types, namely their lowest densities occurring in 2022 (S2 Fig).

Oribatida displayed markedly different dynamics between the two soil types. The only consistent pattern was their negative response to the defaunation treatment. In chernozem soil, total densities varied significantly among years, whereas no clear interannual differences were detected in sandy soil. In contrast, sandy soil showed pronounced seasonal variation, including in *Tectocepheus v. sarekensis* (Fig 4), which did not exhibit seasonal changes in chernozem soil.

## Discussion

Most mite groups recovered rapidly in the defaunated mesocosms during the experimental period. The most straightforward and rapid mechanism of recovery might have been active dispersal from surrounding areas [41]. Fast-dispersing species may have dominated the adjacent grasslands because the grassland had been established only one year prior to the experiment and therefore probably still retained elements of the soil fauna originating from the former agricultural land that had persisted despite the disturbance. Mesostigmata, Tarsonemidae and other Prostigmata (mainly Eupodidae) are known to possess relatively strong active dispersal abilities among mite groups [11,60].

Total densities of Mesostigmata differed little between defaunated and control mesocosms and, in some cases, even exceeded those in the control treatment. This rapid recovery is consistent with previous reports that, although Mesostigmata are sensitive to physical disturbance such as ploughing (e.g., [22]), they are capable of rapid population recovery [23,61].

In particular, a group of Mesostigmata: Gamasina species are frequently reported at high densities in early successional habitats [23,62], consistent with their high mobility and opportunistic life-history strategies. Their rapid recovery is likely facilitated not only by effective dispersal but also by the prompt re-establishment of their prey populations, such as nematodes [25,61]. In addition, some Mesostigmata exhibit rapid reproductive rates characteristic of *r*-strategists [63], although this was not directly assessed in the present study. The observed negative correlation with soil moisture appears to be indirect, reflecting instead a positive association with plant biomass. Mesostigmata were more abundant where grass biomass was higher, which may reflect the more favourable microhabitat conditions associated with taller vegetation, as previously reported for Gamasina mites by Koehler [17].

In addition to rapid dispersal, successful and rapid reproduction in newly colonised habitats is a key component of population recovery following local extinction [32]. Several mite groups exhibited substantially higher densities in defaunated mesocosms than in control mesocosms, indicating that these taxa were able not only to recolonise the habitat rapidly but also to reproduce successfully after establishment. This pattern suggests that these groups possess life-history traits characteristic of r-strategists, including high reproductive rates and rapid population growth [64,65].

Tarsonemidae (Prostigmata), in particular, possess both high reproductive potential and considerable mobility, traits that enhance their ability to exploit newly available habitats [60]. This pattern was evident in our study, where tarsonemid mites reached exceptionally high densities in both control and defaunated mesocosms. These findings indicate that they were able to recolonise defaunated mesocosms rapidly and subsequently increase in abundance to levels comparable to those observed in the controls. They are also known to respond quickly to agricultural disturbance, often exhibiting marked increases in abundance [66].

In chernozem soil, the positive association between Tarsonemidae abundance and plant biomass likely reflects their preference for grass-dominated systems [41,67,68]. The corresponding negative relationship with soil moisture may be an indirect consequence of greater plant biomass, which can enhance evapotranspiration and thereby reduce soil moisture levels. Although Tarsonemidae appear to benefit from increased vegetation, they are reported to be sensitive to heavy grazing [68], which may reduce their populations below a threshold of vegetation cover. Aboveground plant biomass may also influence the quantity and quality of soil microorganisms [69]. As soil-dwelling Tarsonemidae are predominantly fungivorous [70], their abundance is likely mediated indirectly by plant biomass through its effects on the soil microbial community. The absence of a consistent relationship in sandy soil suggests that additional environmental drivers may override the influence of vegetation biomass.

The rapid recovery of Pygmephoroidea populations suggests that these mites also possess effective colonisation abilities. Similar to other heterostigmatid mites such as Tarsonemidae, soil-dwelling Pygmephoroidea are capable of rapid population growth [71], although information on their active dispersal rates remains limited. Nevertheless, our results suggest that Pygmephoroidea may possess dispersal and proliferative capacities similar to those of Tarsonemidae, enabling them to exploit newly available habitats effectively.

Although Pygmephoroidea density was not associated with soil moisture, the weak negative relationship with soil nitrogen content indicates that nutrient availability may influence their populations. Previous studies have reported both positive and negative responses of Pygmephoridae to nitrogen availability [72,73], suggesting that the relationship may depend on the magnitude of nitrogen enrichment or on interacting environmental factors.

The rapid establishment of the populations of the family Iolinidae (Prostigmata) is consistent with effective colonisation of newly available habitats. Their ability to attain high population densities agrees with previous reports describing Iolinidae as dominant members of cropland and grassland mite communities [74], which is consistent with their performance in our study. Certain iolinid species (e.g., *Microtydeus subterraneus, Wood, 1965*) occur in bare sandy soils, exhibit resistance to desiccation, and frequently inhabit the soil surface, where they may disperse by wind [75]. They are also capable of reaching high abundances during early successional stages [17,75]. These traits likely explain why Iolinidae in our study were not strongly dependent on soil moisture and achieved their highest densities in sandy soil.

The positive association between Iolinidae abundance and soil ammonium content suggests that nitrogen availability may indirectly promote their populations by increasing food resources. Although soil algal biomass was not measured, Iolinidae are primarily algivorous [75]. Increased nitrogen availability in originally nutrient-poor habitats, such as sandy soils, may promote algal growth [76], which could in turn support higher abundances of Iolinidae. However, the possibility that increased algal biomass itself contributed to elevated nitrogen availability cannot be excluded. Similarly, Russell and Alberti [77] reported higher densities of *Microtydeus* spp. in sand dunes with elevated total nitrogen content compared to low-nitrogen plots. These findings suggest that nitrogen-driven increases in algal resources may have promoted Iolinidae proliferation in our mesocosms. Nevertheless, further investigation is required to clarify the ecological role and population dynamics of this rapidly growing and abundant group in agricultural systems.

Other Prostigmata comprised several taxa, including Eupodidae, Eriophyoidea, Tydeoidea, and Rhagidiidae; however, specimen numbers were low, and species-level identification was beyond our expertise. Among these groups, Eupodidae are known to have a high potential for rapid active recolonisation [7]. In most cases, densities within “other Prostigmata” group were similar in defaunated and control mesocosms. This pattern is not unexpected, as Eupodidae are highly mobile [11], and many prostigmatid mites are small-bodied with high reproductive potential, enabling rapid recovery even in strongly disturbed or ploughed agricultural soils [20,78].

Some mite groups exhibited lower recovery capacity, characterised by limited active dispersal and slower reproduction. The slower recovery of Endeostigmata suggests that, although these mites were capable of colonising newly available habitats, their population growth was more limited than that of several other mite groups. Endeostigmatid mites are frequently reported from habitats characterised by low organic matter and early successional stages, such as glacier forelands [79] and abandoned open-cast mines [80], indicating strong colonisation ability. They are also common in sandy habitats with warm, dry climates [81,82] and in agricultural soils with low organic matter content [83], indicating that their colonisation ability may depend on environmental conditions and the availability of suitable resources. In arable systems, higher abundances have been recorded in summer than in autumn, possibly in relation to soil moisture and nitrogen availability [84]. The positive associations observed between Endeostigmata abundance, soil moisture, and soil nitrate content in chernozem soil are consistent with this interpretation and suggest that these environmental factors may promote population development. Soil water availability may be particularly important for this group, potentially influencing reproductive processes in mesocosms [85], whereas the negative relationship with aboveground plant biomass may reflect indirect effects of vegetation on habitat conditions or resource availability.

Oribatid mites exhibited the greatest and most persistent differences between defaunated and control mesocosms, with incomplete recovery even eight months after defaunation. Among the mite groups, Oribatida are generally characterised by low recovery potential, reflecting limited dispersal capacity, slow reproductive rates, and high sensitivity to disturbance [18].

The slow recovery observed here is likely attributable primarily to their low mobility [25]. Dispersal in oribatid mites is predominantly ground-based rather than wind-mediated [40], as species inhabiting arboreal habitats are more likely to disperse by wind than soil-dwelling species [39]. Moreover, above-ground movement appears to be more important than below-ground dispersal pathways [33]. In our study, no particular species exhibited disproportionately high colonisation rates; rather, species entered the defaunated mesocosms in proportions similar to those recorded in the controls in both soil types. This suggests that colonisation reflected relative abundance in the surrounding grassland rather than species-specific differences in dispersal ability.

Among the mite groups, oribatid mites showed a distinct response, with recovery patterns differing markedly between soil types. They were largely unable to recolonise defaunated sandy mesocosms, with the exception of October 2022, for which no clear explanation is available. In contrast, recolonisation was consistently more pronounced in chernozem soil across all sampling periods. Baseline differences between the two soil types may have influenced recovery. Species richness was lower in sandy soil than in chernozem soil from the outset. Although both sites were formerly agricultural fields, chernozem soil had higher carbon (humus), nitrogen, and moisture content which can support higher species richness. In addition, soil structure is known to play a key role in shaping soil mite communities, particularly through its influence on pore architecture [86,87]. Sandy soils generally possess lower total pore space than clay-rich soils [88], which can limit habitat availability. Moreover, sandy soils tend to support smaller-bodied species [89] and overall lower soil organism abundance, partly due to reduced water retention [90]. These edaphic constraints likely contributed to the limited recolonisation and lower diversity of Oribatida observed in sandy mesocosms. However, it should be noted that the two soil types were collected from different locations and therefore differed not only in their edaphic properties but also in their original faunal communities. Consequently, the observed differences in recovery patterns cannot be attributed exclusively to soil type.

The contrasting recovery patterns between the two soil types are particularly noteworthy given that they share a relatively abundant species. *T. v. sarekensis* was dominant in both soil types, but reached higher relative abundance in the chernozem soil. This may reflect the stronger legacy of agricultural management at the chernozem site, as *Tectocepheus* spp. are often abundant in croplands [91]. The species is known to recover following disturbance events such as flooding [92], and its surface-dwelling habit and relatively good dispersal ability [39] facilitate colonisation of pioneer habitats [33,90]. Although *T. v. sarekensis* and *T. v. velatus* (Michael, 1880; another subspecies of *Tectocepheus* genus) exhibit broad ecological tolerance and high fecundity [33,93], *T. v. sarekensis* was nevertheless unable to establish and proliferate effectively in defaunated sandy mesocosms, underscoring the constraining influence of edaphic conditions on recolonisation success.

The low recovery rates may also be explained by the slow reproductive rates of oribatid mites [18]. In chernozem soil, the oribatid assemblage comprised a higher proportion of sexually reproducing species than in sandy soil. However, sexually reproducing taxa did not dominate during recolonisation of the defaunated mesocosms. Instead, the parthenogenetic species*, T. v. sarekensis* proved to be one of the most successful recolonisers, with numerous juveniles recorded, indicating both moderately effective colonisation and local reproduction. Although asexual reproduction does not necessarily confer higher colonisation rates in newly available habitats [18], sexually reproducing species have in some cases been shown to colonise more rapidly than parthenogenetic taxa [29,33]. Conversely, parthenogenetic species may exhibit faster population growth following disturbance [61]. In our study, sandy soil was dominated almost exclusively by parthenogenetic species, yet recolonisation success remained limited. Overall, recolonisation in chernozem soil involved several species, but parthenogenetic taxa, particularly *T. v. sarekensis*, contributed most to total oribatid density in defaunated mesocosms, a pattern also observed in the controls.

*Sellnickochthonius immaculatus* (Forsslund, 1942), the other most abundant oribatid species in sandy soil, and *T. v. sarekensis*, frequently co-occur in early successional habitats as pioneer species [18,89,90,94]. Both species are panphytophagous and reproduce parthenogenetically [45,46]. Despite these shared traits, ecological differences between the two species are apparent. *T. v. sarekensis* is a relatively larger, well-sclerotised opportunistic species [18,95] that can attain high densities in arable fields [31,91,96], whereas *S. immaculatus* is generally less abundant in such systems, although it may reach high densities in grasslands and meadows [97,98]. These ecological differences may explain the lower recolonisation ability observed for *S. immaculatus*.

## Conclusions

Our study demonstrates that soil mite communities can recover rapidly following complete defaunation at a fine spatial scale, but recovery is highly taxon-specific rather than uniform across the community. Recovery patterns also differed between soil types, highlighting the importance of local environmental conditions in shaping post-disturbance community reassembly. These findings show that soil mite assemblages should not be regarded as functionally homogeneous and that taxon-specific responses need to be considered when assessing soil resilience and ecosystem recovery. By improving our understanding of the processes underlying soil faunal recovery, this study also provides a useful framework for interpreting the effects of disturbance in soil monitoring and ecological restoration studies.

## Supporting information

S1 Fig Photos of the experimental design and the interior of the mesocosms with grass plants. (DOCX)

S2 Fig (8 figures on 4 pages): Density of different mite groups (per 400 cm^3^ sample; 50 cm^2^ area) in the two soil types across three years and two months (July: summer and October: autumn). Letters indicate significant differences within each year among treatment × month combinations, based on gls models (S2 Table). The scale of the y-axis is different for the Pygmephoroidea between the two soil types. Number of replicates in a given month: N(2021)=16, N(2022,2023)=10.
(DOCX)

S3 Fig Rate of juveniles and parthenogenetic species among oribatid mites in the two soil types across three years and two months (July: summer and October: autumn). Number of replicates in a given month: N(2021)=16, N(2022,2023)=10.
(DOCX)

S1 Table Summary of linear models used in SEM analysis. (DOCX)

S2 Table F- and p-values from linear (lm) and generalized least squares (gls) models of mite group densities (log(x + 1)), with treatment and month (season) included as separate factors for each of the three years. The last three columns show the three model types used for 2021, 2022, and 2023.
(DOCX)

S3 Table F- and p-values of factors and environmental variables from redundancy analyses conducted on the full dataset (all years combined), as well as separately for each year and for each soil type. All the models were also run without the factors. Significant parameters (in models without factors) are shown with asterisks, *p < 0.05, **p < 0.01, ***p < 0.001. (SIR = substrate-induced respiration).
(DOCX)

S4 Table Mean density (individuals /m^2^) of Oribatida species in the two soil types in control mesocosms. (DOCX)

S5 Table Trophic guilds and reproduction modes of oribatid mites. (DOCX)

S6 Table Mean (± SD) values of soil environmental variables for the two soil types. Lowercase letters indicate significant differences among treatment levels and month (seasons) within a given year. (SIR = substrate induced respiration).
(DOCX)
