# Supplementary material for: Fine-scale recovery of mite communities following local extinctions on grasslands
Source: PLoS One. 2026 Sep 1;21(9):e0357403. doi: 10.1371/journal.pone.0357403 (PMC13533364; doi:10.1371/journal.pone.0357403)
Supplement: Supplementary file 2 [file pone.0357403.s002.docx]

**S2 Fig. (8 figures on 4 pages): Density of different mite groups (per 400 cm³ sample; 50 cm² area) in the two soil types across three years and two months (July: summer and October: autumn).** Letters indicate significant differences within each year among treatment × month combinations, based on gls models (S2 Table). The scale of the y-axis is different for the Pygmephoroidea between the two soil types. Number of replicates in a given month: N(2021)=16, N(2022,2023)=10.
