# Supplementary material for: Fine-scale recovery of mite communities following local extinctions on grasslands
Source: PLoS One. 2026 Sep 1;21(9):e0357403. doi: 10.1371/journal.pone.0357403 (PMC13533364; doi:10.1371/journal.pone.0357403)
Supplement: Supplementary file 3 [file pone.0357403.s003.docx]

**S3 Fig. Rate of juveniles and parthenogenetic species among oribatid mites in the two soil types across three years and two months (July: summer and October: autumn).** Number of replicates in a given month: N(2021)=16, N(2022,2023)=10.
