# Supplementary material for: Fine-scale recovery of mite communities following local extinctions on grasslands
Source: PLoS One. 2026 Sep 1;21(9):e0357403. doi: 10.1371/journal.pone.0357403 (PMC13533364; doi:10.1371/journal.pone.0357403)
Supplement: Supplementary file 4 [file pone.0357403.s004.docx]

**S1 Table Summary of linear models used in SEM analysis.**

**Chernozem soil:**

model1: lm(Tarsonemidae ~ month+ year + treatment+ soil moisture + plant biomass +Total_N_soil+

NH4_Soil + NO3_Soil, data=data)

model2 <- lm(Pygmephoroidae ~	month +Treatment+ year + Total_N_soil,

data=data)

model3 <- lm(Mesostigmata ~ Treatment +year + month+ Soil_moisture + Total_N_soil + NH4 Soil+

Grass weight+ Tarsonemidae+ Pygmephoroidae, data=data)

model4 <- lm(Total_N_soil ~ Treatment + month + year + Soil_moisture+ NO3_Soil + NH4_Soil , data=data)

model5 <- lm(Iolidae ~ year +month +Treatment+NH4_Soil + Soil_moisture+ Total_N_soil+ Tarsonemidae, data=data)

model6 <- lm(Oribatida ~ month + Treatment +year+ Soil_moisture+ NH4_Soil+Total_N_soil, data=data)

model7 <- lm(Prostigmata ~ month + year + Treatment + NO3_Soil + Soil_moisture+ NH4_Soil+Total_N_soil+ Oribatida + Iolidae+ Tarsonemidae, data=data)

model8 <- lm(Endeostigmata ~ month + year + Treatment + Grass_weight + NO3_Soil+ Iolidae+ Prostigmata+ Pygmephoroidae, data=data)

model9 <- lm(Soil_moisture ~ Treatment + month + year, data=data)

model10 <- lm(Grass_weight ~ Treatment + month + year + Soil_moisture + Total_N_soil+ NH4_Soil data=data)

model11 <- lm(NO3_Soil ~ Treatment + month + year + Soil_moisture, data=data)

model12 <- lm(NH4_Soil ~ Treatment + month + year + Soil_moisture, data=data)

**Sandy soil:**

model1 <- lm(Tarsonemidae ~ month+ year +Treatment+ Soil_moisture + Total_N_soil, data=data)

model2 <- lm(Pygmephoroidae ~ month +Treatment+ year + Soil_moisture + NO3_Soil+NH4_Soil+Total_N_soil+ Grass_weight, data=data)

model3 <- lm(Mesostigmata ~ Treatment +year + month+ Soil_moisture + Total_N_soil + NH4_Soil + Grass_weight, data=data)

model4 <- lm(Total_N_soil ~ Treatment + month + year + Soil_moisture+ NO3_Soil + NH4_Soil ,

model5 <- lm(Iolidae ~	year +month +Treatment+NH4_Soil +NO3_Soil, data=data)

model6 <- lm(Oribatida ~ month + Treatment + Grass_weight +NO3_Soil + Iolidae + Tarsonemidae, data=data)

model7 <- lm(Prostigmata ~ month + year + Treatment + NO3_Soil + Soil_moisture+ Total_N_soil

+ Tarsonemidae + NH4_Soil, data=data)

model8 <- lm(Endeostigmata ~ year + Treatment + Soil_moisture + Grass_weight + NH4_Soil+Total_N_soil+ Iolidae+ Oribatida, data=data)

model9 <- lm(Soil_moisture ~ Treatment + month + year, data=data)

model10 <- lm(Grass_weight ~ Treatment + month + year + Soil_moisture + Total_N_soil+ NH4_Soil, data=data)

model11 <- lm(NO3_Soil ~ Treatment + month + year + Soil_moisture, data=data)

model12 <- lm(NH4_Soil ~ Treatment + month + year + Soil_moisture, data=data)
