# Supplementary material for: Fine-scale recovery of mite communities following local extinctions on grasslands
Source: PLoS One. 2026 Sep 1;21(9):e0357403. doi: 10.1371/journal.pone.0357403 (PMC13533364; doi:10.1371/journal.pone.0357403)
Supplement: Supplementary file 5 [file pone.0357403.s005.docx]

**S2 Table F- and p-values from linear (lm) and generalized least squares (gls) models of mite group densities (log(x + 1)), with treatment and month (season) included as separate factors for each of the three years.** The last three columns show the three model types used for 2021, 2022, and 2023.

|  | 2021 | df=60 |  |  |  |  | 2022 | df=36 |  |  |  |  | 2023 | df=35 |  |  |  |  |  |  |  |
| --- | --- | --- | --- | --- | --- | --- | --- | --- | --- | --- | --- | --- | --- | --- | --- | --- | --- | --- | --- | --- | --- |
|  | Treatment | | month | | interaction | | Treatment | | month | | interaction | | Treatment | | month | | interaction | |  |  |  |
|  | F | p | F | p | F | p | F | p | F | p | F | p | F | p | F | p | F | p |  |  |  |
| **Chernozem** |  |  |  |  |  |  |  |  |  |  |  |  |  |  |  |  |  |  |  |  |  |
| Mesostigmata | 1.87 | 0.18 | 0.12 | 0.73 | 3.17 | 0.08 | 1.08 | 0.306 | 13.32 | 8E-04 | 1.98 | 0.168 | 58.9 | <.0001 | 39.5 | <.0001 | 0.014 | 0.905 | lm | lm | lm |
| Endeostigmata | 151 | <.0001 | 83.9 | <.0001 | 1.65 | 0.2 | 1.16 | 0.352 | 6.06 | 0.019 | 0.25 | 0.622 | 34.7 | <.0001 | 0.001 | 0.971 | 8.2 | 0.007 | gls | lm | lm |
| Oribatida | 137 | <.0001 | 0.02 | 0.883 | 1.19 | 0.279 | 95.2 | <.0001 | 12.89 | <.0001 | 15.82 | <.0001 | 39.54 | <.0001 | 1.96 | 0.169 | 6.46 | 0.016 | gls | lm | lm |
| Tarsonemidae | 0.002 | 0.959 | 0.822 | 0.368 | 2.096 | 0.153 | 3.19 | 0.082 | 4.44 | 0.042 | 0.876 | 0.356 | 0.008 | 0.929 | 2.47 | 0.125 | 0.236 | 0.63 | gls | gls | lm |
| Pygmephoroidea | 0.69 | 0.411 | 6.77 | 0.012 | 14.86 | 0.0003 | 12.25 | 0.001 | 3.32 | 0.077 | 3.64 | 0.113 | 9.22 | 0.005 | 0.02 | 0.891 | 11.83 | 0.002 | lm | lm | gls |
| Iolinidae | 67.3 | <.0001 | 1.95 | <.0001 | 1.33 | 0.254 | 0.032 | 0.86 | 0.32 | 0.575 | 0.417 | 0.522 | 20.76 | <.0001 | 2.73 | 0.107 | 0.56 | 0.459 | lm | lm | lm |
| Other Prostigmata | 4.36 | 0.041 | 45.6 | <.0001 | 24.36 | <.0001 | 0.23 | 0.636 | 2.1 | 0.156 | 0.065 | 0.8 | 3.15 | 0.084 | 29.09 | <.0001 | 7.32 | 0.01 | lm | gls | gls |
|  |  |  |  |  |  |  |  |  |  |  |  |  |  |  |  |  |  |  |  |  |  |
| **Sandy soil** |  |  |  |  |  |  |  |  |  |  |  |  |  |  |  |  |  |  |  |  |  |
| Mesostigmata | 10.32 | 0.002 | 9.96 | 0.002 | 4.24 | 0.044 | 16.11 | 3E-04 | 1.54 | 0.223 | 1.45 | 0.237 | 5.36 | 0.027 | 215.1 | <.0001 | 17.37 | 2E-04 | lm | lm | gls |
| Endeostigmata | 131 | <.0001 | 52.4 | <.0001 | 12.44 | 8E-04 | 5.11 | 0.03 | 0.14 | 0.715 | 1.35 | 0.253 | 27.1 | <.0001 | 0.49 | 0.489 | 0.49 | 0.489 | gls | lm | lm |
| Oribatida | 330 | <.0001 | 15.9 | 2E-04 | 11.66 | 0.001 | 21.97 | <.0001 | 7.4 | 0.01 | 0.347 | 0.56 | 430 | <.0001 | 1.38 | 0.248 | 10.47 | 0.003 | gls | gls | gls |
| Tarsonemidae | 20.07 | <.0001 | 29.6 | <.0001 | 1.09 | 0.301 | 6.61 | 0.015 | 7.02 | 0.012 | 1.58 | 0.217 | 16.88 | 2E-04 | 1.66 | 0.207 | 2.12 | 0.154 | gls | lm | lm |
| Pygmephoroidea | 14.08 | 4E-04 | 44.4 | <.0001 | 6.07 | 0.017 | 1.036 | 0.316 | 56.1 | <.0001 | 0.21 | 0.648 | 28.03 | <.0001 | 10.97 | 0.002 | 0.187 | 0.668 | gls | lm | lm |
| Iolinidae | 138 | <.0001 | 26 | <.0001 | 11.8 | 0.001 | 41.5 | <.0001 | 302 | <.0001 | 9.16 | 0.005 | 31.1 | <.0001 | 68.2 | <.0001 | 0.05 | 0.819 | gls | gls | gls |
| Other Prostigmata | 20.28 | <.0001 | 1.92 | 0.171 | 2.47 | 0.121 | 13.67 | 7E-04 | 1.3 | 0.263 | 6.51 | 0.016 | 14.32 | 6E-04 | 2.72 | 0.108 | 1.98 | 0.168 | lm | gls | lm |
