# Supplementary material for: Fine-scale recovery of mite communities following local extinctions on grasslands
Source: PLoS One. 2026 Sep 1;21(9):e0357403. doi: 10.1371/journal.pone.0357403 (PMC13533364; doi:10.1371/journal.pone.0357403)
Supplement: Supplementary file 6 [file pone.0357403.s006.docx]

**S3 Table F- and p-values of factors and environmental variables from redundancy analyses conducted on the full dataset (all years combined), as well as separately for each year and for each soil type.** All the models were also run without the factors. Significant parameters (in models without factors) are shown with asterisks, *p < 0.05, **p < 0.01, ***p < 0.001. (SIR= substrate-induced respiration)

+++: variables measured only in 2021 and 2022; therefore, these variables were excluded from the overall (full) model.

| RDA |  | full |  | without factors | 2021 |  | without factors | 2022 |  | without factors | 2023 |  | without factors |
| --- | --- | --- | --- | --- | --- | --- | --- | --- | --- | --- | --- | --- | --- |
|  | **Sandy soil** | F | p |  | F | p |  | F | p |  | F | p |  |
|  | R2 | 0.546 |  |  | 0.437 |  |  | 0.67 |  |  | 0.382 |  |  |
|  | Total | 17.366 | **0.001** |  | 7.0107 | 0.001 |  | 10.128 | 0.001 |  | 3.7822 | 0.001 |  |
| factors | year | 19.1637 | **0.001** |  |  |  |  |  |  |  |  |  |  |
|  | month | 33.4508 | **0.001** |  | 3.4591 | **0.016** |  | 15.0537 | **0.001** |  | 2.1293 | 0.074 |  |
|  | treatment | 13.7528 | **0.001** |  | 8.5095 | **0.001** |  | 0.8605 | 0.413 |  | 4.2016 | **0.006** |  |
| variables | Soil_moisture | 6.4488 | **0.001** | *** | 0.8896 | 0.463 | ** | 2.0997 | 0.119 | * | 0.6951 | 0.619 | ** |
|  | Plant biomass | 4.4547 | **0.004** | ** | 3.3989 | **0.018** | ** | 0.4293 | 0.659 |  | 1.6541 | 0.138 |  |
|  | Total_N_soil | 1.3009 | 0.237 | *** | 2.045 | 0.082 |  | 1.8104 | 0.161 |  | 0.7472 | 0.572 |  |
|  | NH_4_^+^-N soil | 1.9981 | 0.078 | *** | 2.765 | **0.032** | ** | 0.4982 | 0.639 | ** | 0.8048 | 0.522 |  |
|  | NO_3_^-^-N soil | 4.3727 | **0.005** | * | 0.7799 | 0.529 |  | 1.2828 | 0.288 |  | 3.4899 | **0.012** | * |
|  | total_N_Plant | 0.5331 | 0.72 | *** | 0.7351 | 0.575 |  | 1.5651 | 0.201 |  | 0.29 | 0.917 |  |
|  | Humus%+++ | 0.7912 | 0.516 |  | 0.6147 | 0.681 |  | 0.5702 | 0.553 |  |  |  |  |
|  | SIR+++ | 0.7855 | 0.531 |  | 0.5079 | 0.76 |  | 0.2481 | 0.818 |  |  |  |  |
|  |  |  |  |  |  |  |  |  |  |  |  |  |  |
|  |  | full |  | without factors | 2021 |  | without factors | 2022 |  | without factors | 2023 |  | without factors |
|  | **Chernozem soil** | F | p |  | F | p |  | F | p |  | F | p |  |
|  | R2 | 0.319 |  | 0.189 | 0.247 |  | 0.153 | 0.289 |  |  | 0.278 |  | 0.098 |
|  | Total | 7.6497 | 0.001 | 6.5137*** | 3.5898 | 0.001 | 2.8946*** | 2.9881 | 0.001 | 0.087 | 2.8285 | 0.001 | 1.687* |
| factors | year | 5.2656 | **0.001** |  |  |  |  |  |  | 1.6219(ns) | |  |  |
|  | month | 4.9015 | **0.001** |  | 3.3708 | **0.008** |  | 4.5064 | **0.006** |  | 1.896 | 0.124 |  |
|  | treatment | 12.8001 | **0.001** |  | 5.7533 | **0.001** |  | 6.9238 | **0.001** |  | 6.681 | **0.002** |  |
| variables | Soil_moisture | 1.9947 | 0.076 | *** | 0.6118 | 0.708 | * | 1.9366 | 0.103 |  | 1.9066 | 0.119 |  |
|  | Plant biomass | 2.7369 | **0.023** | *** | 1.8323 | 0.111 | * | 1.4276 | 0.225 |  | 0.4435 | 0.821 | ** |
|  | Total N soil | 1.7159 | 0.124 | * | 2.1355 | 0.067 | *** | 0.7711 | 0.577 |  | 0.8194 | 0.472 |  |
|  | NH_4_^+^-N soil | 0.626 | 0.717 | * | 0.3233 | 0.905 |  | 0.7719 | 0.57 |  | 0.4089 | 0.806 |  |
|  | NO_3_^-^-N soil | 1.5578 | 0.151 |  | 1.1955 | 0.293 |  | 0.6234 | 0.667 |  | 0.7257 | 0.57 |  |
|  | total_N_Plant | 0.5397 | 0.768 | * | 0.4115 | 0.849 |  | 0.8862 | 0.485 |  | 0.2604 | 0.924 |  |
|  | Humus%+++ | 1.3659 | 0.233 |  | 1.5151 | 0.193 |  | 2.5404 | **0.038** |  |  |  |  |
|  | SIR+++ | 1.4674 | 0.157 |  | 0.7495 | 0.601 |  | 0.8803 | 0.501 |  |  |  |  |
