# Supplementary material for: Fine-scale recovery of mite communities following local extinctions on grasslands
Source: PLoS One. 2026 Sep 1;21(9):e0357403. doi: 10.1371/journal.pone.0357403 (PMC13533364; doi:10.1371/journal.pone.0357403)
Supplement: Supplementary file 7 [file pone.0357403.s007.docx]

**S4 Table Mean density (individuals /m^2^) of Oribatida species in the two soil types in control mesocosms.**

|  |  | Mean number/ m^2^ | | | | | |
| --- | --- | --- | --- | --- | --- | --- | --- |
|  |  | SANDY | | | CHERNOZEM | | |
|  |  | **2021** | **2022** | **2023** | **2021** | **2022** | **2023** |
| dominant | *Tectocepheus velatus sarekensis (Trägardh, 1910)* | 452 | 728 | 800 | 1275 | 1765 | 1432 |
|  | *Tectocepheus juveniles* | 794 | 1278 | 2526 | 3769 | 2505 | 3621 |
|  | *Sellnickochthonius immaculatus (Forsslund, 1942)* | 5335 | 4433 | 3747 | 31 | 70 | 42 |
|  | Brachychthoniidae juveniles | 2897 | 694 | 2084 | 0 | 0 | 0 |
| only chernozem soil | *Scheloribates laevigatus (C.L. Koch, 1836)* | 0 | 0 | 0 | 256 | 965 | 368 |
|  | *Ramusella elliptica (Berlese, 1908)* | 0 | 0 | 0 | 469 | 480 | 189 |
|  | *Epilohmannia cylindrica (Berlese, 1904)* | 0 | 0 | 0 | 50 | 530 | 253 |
|  | *Graptoppia paraanalis (Subias & Rodriguez, 1985)* | 0 | 0 | 0 | 306 | 115 | 347 |
|  | *Oribatula pannonica (Willmann, 1949)* | 0 | 0 | 0 | 306 | 215 | 137 |
|  | *Corynoppia kosarovi (Jeleva, 1962)* | 0 | 0 | 0 | 219 | 205 | 158 |
|  | *Oppiella (Rhinoppia) obsoleta (Paoli, 1908)* | 0 | 0 | 0 | 206 | 75 | 263 |
|  | *Scheloribates fimbriatus (Thor, 1930)* | 0 | 0 | 0 | 13 | 260 | 32 |
|  | *Protoribates capucinus (Berlese, 1908)* | 0 | 0 | 0 | 50 | 105 | 147 |
|  | *Hypochthonius luteus (Oudemans, 1917)* | 0 | 0 | 0 | 156 | 5 | 95 |
| only sandy soil | *Brachychthonius pius (Moritz, 1976)* | 155 | 178 | 21 | 0 | 0 | 0 |
|  | *Sellnickochthonius oesziae (Balogh & Mahunka, 1979)* | 155 | 117 | 74 | 0 | 0 | 0 |
| both of the soil types | *Rhysotritia ardua (C.L. Koch, 1841)* | 277 | 122 | 95 | 13 | 45 | 32 |
|  | *Microppia minus (Paoli, 1908)* | 26 | 39 | 11 | 25 | 5 | 0 |
|  | *Adelphacarus sellnicki (Grandjean, 1952)* | 19 | 0 | 0 | 31 | 0 | 0 |
|  | other oribatid juveniles | 19 | 11 | 0 | 1638 | 3250 | 1621 |
