# Supplementary material for: Fine-scale recovery of mite communities following local extinctions on grasslands
Source: PLoS One. 2026 Sep 1;21(9):e0357403. doi: 10.1371/journal.pone.0357403 (PMC13533364; doi:10.1371/journal.pone.0357403)
Supplement: Supplementary file 8 [file pone.0357403.s008.docx]

**S5 Table Trophic guilds and reproduction modes of oribatid mites.**

|  | Reproduction mode | Source | | Size | Feeding guild**** | | |
| --- | --- | --- | --- | --- | --- | --- | --- |
| *Tectocepheus velatus sarekensis (Trägardh, 1910)* | asexual | Maraun et al., 2022 | | large | 2 | Schneider et al., 2004 | |
| *Tectocepheus juveniles* | asexual | Maraun et al 2022 | | large | 2 |  |  |
| *Sellnickochthonius immaculatus (Forsslund, 1942)* | asexual | Maraun et al 2022 | | small | 2 | Farská et al., 2014 | |
| Brachychthoniidae juvenile | asexual | Maraun et al 2022 | | small | 2 |  |  |
| *Scheloribates laevigatus (C.L. Koch, 1836)* | sexual | Maraun et al 2022 | | large | 2 | Farska et al 2014 | |
| *Ramusella elliptica (Berlese, 1908)* | sexual | Maraun et al 2022 | | small | 2 | Siepel and Ruiter-Dijkman, 1993 | |
| *Epilohmannia cylindrica (Berlese, 1904)* | asexual | Maraun et al 2022 | | large | 3 | Pan et al., 2022 | |
| *Graptoppia paraanalis (Subias & Rodriguez, 1985)* | asexual | inference *** | | small | 2 | inference |  |
| *Oribatula pannonica (Willmann, 1949)* | sexual | Maraun et al 2022 | | large | 2 | Schneider et al 2004 | |
| *Corynoppia kosarovi (Jeleva, 1962)* | sexual | sex ratio* | | small** | 2 | inference |  |
| *Oppiella (Rhinoppia) obsoleta (Paoli, 1908)* | sexual | Farska et al 2014 | | small | 1 | Schneider et al 2004 | |
| *Scheloribates fimbriatus (Thor, 1930)* | sexual | Maraun et al 2022 | | large | 2 | Farska et al 2014 | |
| *Protoribates capucinus (Berlese, 1908)* | asexual | Maraun et al 2022 | | large | 2 | Pan et al 2022 | |
| *Hypochthonius luteus (Oudemans, 1917)* | asexual | Maraun et al 2022 | | large | 1 | Schneider et al 2004 | |
| *Brachychthonius pius (Moritz, 1976)* | asexual | Maraun et al 2022 | | small | 2 | Farska et al 2014 | |
| *Sellnickochthonius oesziae (Balogh & Mahunka, 1979)* | asexual | inference | | small | 2 | Farska et al 2014 | |
| *Rhysotritia ardua (C.L. Koch, 1841)* | asexual | Maraun et al 2022 | | large | 3 | Schneider et al 2004 | |
| *Microppia minus (Paoli, 1908)* | asexual | Maraun et al 2022 | | small | 1 | Haynert et al., 2017 | |
| *Adelphacarus sellnicki (Grandjean, 1952)* | sexual | Lotfollahi et al., 2017 | | large | ? |  |  |
| *Sex ratio was determined in three samples and the rate of males were the followings: 0.73 (11 specimens), 0.67 (12 specimens), 0.75 (4 specimens). | | |  |  |  |  |  |
| **According to Akrami, 2015 |  |  |  |  |  |  |  |
| ***** According to Cianciolo and Norton, 2006  ****** Omnivores (1): primarily feeding on fungi and/or living or dead animals;  Secondary decomposers (2): mainly feeding on fungi, with partial consumption of plant material;  Primary decomposers (3): predominantly consuming plant parts and litter. | | | | | | | |
