# Supplementary material for: Fine-scale recovery of mite communities following local extinctions on grasslands
Source: PLoS One. 2026 Sep 1;21(9):e0357403. doi: 10.1371/journal.pone.0357403 (PMC13533364; doi:10.1371/journal.pone.0357403)
Supplement: Supplementary file 9 [file pone.0357403.s009.docx]

**S6 Table Mean (± SD) values of soil environmental variables for the two soil types.** Lowercase letters indicate significant differences among treatment levels and month (seasons) within a given year. (SIR= substrate-induced respiration)

| **Year** | **Month** | **Treatment** | **Soil moisture** | | **Plant biomass** |  | **Total N content** | | **Soil NH4^+^-N** |  | **Soil NO3^-^-N** |  | **Plant N content** | | **Humus** |  | **SIR** |  |
| --- | --- | --- | --- | --- | --- | --- | --- | --- | --- | --- | --- | --- | --- | --- | --- | --- | --- | --- |
| **SANDY SOIL** |  |  | **V/V%** |  | **g/mesocosm** |  | **g/kg** |  | **mg/kg** |  | **mg/kg** |  | **g/kg** |  | **g/kg** |  | **µg C-CO2 /g soil /h** | |
| 2021 | July | control | 13.95 ± 0.95 | a | 3.61 ± 1.56 | a | 1.1 ± 0.0 | a | 2.00 ± 0.45 | a | 4.17 ± 2.51 | a | 24.1 ± 2.8 | a | 17.5 ± 0.8 | a | 5.84 ± 1.35 | a |
| 2021 | July | defaunated | 13.22 ± 1.21 | a | 14.83 ± 7.74 | b | 1.1 ± 0.0 | a | 1.76 ± 0.41 | a | 3.55 ± 2.77 | ab | 21.3 ± 2.1 | b | 17.4 ± 1.1 | a | 5.30 ± 1.22 | ab |
| 2021 | October | control | 2.91 ± 1.17 | b | 12.53 ± 2.83 | b | 1.3 ± 0.1 | b | 1.23 ± 0.55 | b | 2.28 ± 0.94 | b | 18.8 ± 1.7 | c | 15.6 ± 0.8 | b | 5.44 ± 0.98 | ab |
| 2021 | October | defaunated | 4.16 ± 1.36 | c | 11.7 ± 5.03 | b | 1.3 ± 0.1 | b | 0.87 ± 0.20 | b | 2.7 ± 1.36 | ab | 17.3 ± 1.9 | c | 15.6 ± 1.1 | b | 4.60 ± 0.89 | b |
| Used model type | | | lm |  | gls |  | lm |  | gls |  | gls |  | lm |  | lm |  | lm |  |
| 2022 | July | control | 2.68 ± 1.29 | a | 23.56 ± 7.88 | a | 2.3 ± 0.1 | a | 1.92 ± 0.47 | a | 3.23 ± 4.2 | ab | 14.5 ± 1.4 | a | 14.1 ± 0.9 | a | 3.71 ± 2.62 | a |
| 2022 | July | defaunated | 2.01 ± 0.37 | a | 14.09 ± 6.54 | b | 1.6 ± 0.6 | b | 1.63 ± 0.45 | ab | 1.35 ± 0.45 | a | 13.5 ± 1.0 | a | 15.3 ± 0.5 | b | 4.68 ± 1.09 | a |
| 2022 | October | control | 4.86 ± 1.32 | b | 8.07 ± 5.3 | b | 1.23 ± 0.05 | b | 2.78 ± 1.04 | bc | 4.2 ± 5.5 | ab | 11.6 ± 10.0 | a | 14.9 ± 1.0 | ab | 4.72 ± 0.56 | a |
| 2022 | October | defaunated | 2.82 ± 0.92 | a | 8.51 ± 3.61 | b | 1.26 ± 0.04 | b | 3.24 ± 0.32 | c | 2.71 ± 1.42 | b | 12.5 ± 7.3 | a | 15.1 ± 0.7 | b | 5.13 ± 0.51 | a |
| Used model type | | | gls |  | lm |  | gls |  | gls |  | gls |  | gls |  | lm |  | gls |  |
| 2023 | July | control | 4.88 ± 1.41 | a | 3.64 ± 2.84 | a | 1.20 ± 0.09 | a | 2.98 ± 0.74 | a | 4.21 ± 1.22 | a | 25.2 ± 4.5 | a | Not measured in this year | | | |
| 2023 | July | defaunated | 2.31 ± 0.61 | b | 16.06 ± 2.23 | b | 1.32 ± 0.04 | bc | 3.58 ± 0.79 | a | 8.72 ± 15.31 | a | 27 ± 5.5 | a |  |  |  |  |
| 2023 | October | control | 10.39 ± 1.3 | c | 4.27 ± 3.38 | a | 1.26 ± 0.06 | ab | 2.86 ± 0.53 | a | 3.76 ± 1.86 | a | 20.9 ± 8.8 | ab |  |  |  |  |
| 2023 | October | defaunated | 8.41 ± 1.45 | d | 14.74 ± 8.23 | b | 1.35 ± 0.04 | c | 2.89 ± 0.61 | a | 4.68 ± 4.31 | a | 13.4 ± 5.9 | b |  |  |  |  |
| Used model type | | | lm |  | gls |  | lm |  | lm |  | lm |  | lm |  |  |  |  |  |

| **Year** | **Month** | **Treatment** | **Soil moisture** |  | **Grass biomass** |  | **Total N content** |  | **Soil NH4^+^-N** |  | **Soil NO3^-^-N** |  | **Plant N content** |  | **Humus%** |  | **SIR** |  |
| --- | --- | --- | --- | --- | --- | --- | --- | --- | --- | --- | --- | --- | --- | --- | --- | --- | --- | --- |
| **CHERN. SOIL** |  |  | **V/V%** |  | **g/mesocosm** | | **g/kg** |  | **mg/kg** |  | **mg/kg** |  | **g/kg** |  | **g/kg** |  | **µg C-CO2 /g soil /h** | |
| 2021 | July | control | 21.67 ± 2.72 | a | 6.29 ± 5.35 | a | 2.0 ± 0.1 | a | 1.47 ± 0.46 | a | 3.12 ± 1.44 | a | 18.2 ± 5.2 | a | 30.9 ± 1.4 | a | 5.54 ± 1.28 | a |
| 2021 | July | defaunated | 30.69 ± 2.92 | b | 13.64 ± 9.93 | bc | 2.0 ± 0.1 | a | 1.35 ± 0.56 | a | 2.7 ± 1.33 | a | 12.9 ± 1.2 | b | 30.7 ± 1.4 | a | 4.98 ± 1.39 | ab |
| 2021 | October | control | 22.41 ± 2.3 | a | 4.43 ± 2.22 | ab | 2.2 ± 0.1 | b | 1.13 ± 0.47 | a | 3.75 ± 6.91 | a | 16.6 ± 3.9 | a | 30.8 ± 1.1 | a | 4.54 ± 0.74 | b |
| 2021 | October | defaunated | 27.76 ± 3.89 | c | 11.08 ± 3.23 | c | 2.2 ± 0.1 | b | 1.11 ± 0.19 | a | 1.14 ± 1.05 | a | 11.0 ± 1.4 | c | 32.5 ± 1.6 | b | 4.7 ± 0.7 | ab |
|  |  |  | lm |  | gls |  | lm |  | lm |  | lm |  | gls |  | lm |  | lm |  |
| 2022 | July | control | 11.34 ± 2.58 | ab | 20.95 ± 11.29 | a | 2.2 ± 0.1 | a | 2.63 ± 0.56 | a | 0.96 ± 0.45 | a | 11.4 ± 0.8 | b | 29.8 ± 1.3 | a | 4.46 ± 1.64 | a |
| 2022 | July | defaunated | 18.34 ± 3.54 | c | 19.01 ± 15.75 | a | 2.7 ± 0.9 | a | 2.47 ± 0.44 | a | 0.78 ± 0.63 | a | 9.2 ± 1.7 | a | 29.5 ± 1.2 | ab | 5.97 ± 1.81 | ab |
| 2022 | October | control | 11.32 ± 2.82 | a | 10.1 ± 6.98 | a | 2.2 ± 0.1 | a | 2.09 ± 0.67 | a | 2.13 ± 1.07 | b | 13.0 ± 5.1 | ab | 31.1 ± 1.3 | b | 5.92 ± 0.69 | ab |
| 2022 | October | defaunated | 14.8 ± 2.45 | b | 11.62 ± 3.37 | a | 2.2 ± 0.1 | a | 2.36 ± 0.64 | a | 2.11 ± 0.75 | b | 12.3 ± 1.4 | b | 30.8 ± 1.2 | ab | 6.17 ± 0.73 | b |
|  |  |  | lm |  | gls |  | gls |  | lm |  | lm |  | gls |  | lm |  | gls |  |
| 2023 | July | control | 9.33 ± 1.58 | a | 8.5 ± 4.63 | a | 2.3 ± 0.1 | b | 4.06 ± 0.48 | a | 2.23 ± 0.82 | a | 14.3 ± 2.4 | a |  | Not measured in this year. | | |
| 2023 | July | defaunated | 7.54 ± 2.01 | a | 27.11 ± 4.49 | b | 2.1 ± 0.1 | a | 3.65 ± 0.35 | a | 1.8 ± 1.26 | a | 15.0 ± 3.0 | a |  |  |  |  |
| 2023 | October | control | 18.39 ± 3.78 | b | 10.56 ± 15.35 | a | 2.3 ± 0.1 | b | 3.95 ± 0.8 | a | 9.31 ± 7.11 | b | 20.0 ± 9.1 | a |  |  |  |  |
| 2023 | October | defaunated | 22.6 ± 4.24 | b | 18.03 ± 6.82 | ab | 2.2 ± 0.1 | ab | 3.8 ± 0.69 | a | 5.01 ± 6.4 | ab | 17.3 ± 4.0 | a |  |  |  |  |
|  |  |  | gls |  | lm |  | lm |  | lm |  | gls |  | gls |  |  |  |  |  |
