# Supplementary material for: Fine-scale recovery of mite communities following local extinctions on grasslands
Source: PLoS One. 2026 Sep 1;21(9):e0357403. doi: 10.1371/journal.pone.0357403 (PMC13533364; doi:10.1371/journal.pone.0357403)
Supplement: Supplementary file 10 [file pone.0357403.s010.docx]

These findings provide valuable insights for researchers studying soil fauna in disturbed agricultural systems, particularly in understanding taxon-specific recovery dynamics and the role of environmental context in shaping resilience. However, the manuscript requires some revisions prior to publication, which are outlined below.

Abstract:

It is recommended to shorten the abstract, particularly by simplifying some of the long sentences, which would improve its clarity and enhance readability for a broader audience. In its current form, the abstract is somewhat information-dense, which may make it more difficult to quickly grasp the main results of the study.

Intorduction:

Lines 88-99: The paragraph addressing disturbances to soil fauna communities largely repeats concepts that have already been introduced earlier in the introduction, particularly regarding the effects of agricultural practices, spatial patchiness, and biodiversity loss. To improve the overall clarity and conciseness of the manuscript, this section could be merged with the preceding discussion and more tightly integrated. A more compact formulation would help avoid redundancy and strengthen the logical flow of the argument.

Additionally, the concepts of resilience and recovery are repeated in several parts of the introduction, leading to redundancy. It is recommended to consolidate this idea into a single, more concise section to improve clarity and coherence of the text.

Lines 110-115: The hypotheses are clearly formulated and relevant to the study objectives. However, the authors may consider strengthening this section by supporting the individual hypotheses with appropriate citations from the existing literature. This would help further contextualize the predictions and better justify their theoretical basis.

Materials and methods

Lines 118-124: The authors should consider adding an appropriate citation for the WRB soil classification to ensure proper referencing of the soil taxonomy used. In addition, the citation format (Gergócs et al., 2022) should be revised to ensure consistency with the citation style used throughout the manuscript.

Lines 159-160: The list of plant species would benefit from standardisation by adding the taxonomic authority to the Latin names (e.g. *Festuca rubra* L.). This would ensure consistency with botanical nomenclature conventions and improve taxonomic precision throughout the manuscript.

Lines 177-198: The Methods section would benefit from clearer structuring. In particular, it is recommended to separate the methodological description into distinct subsections for biological analyses and chemical/physicochemical analyses. This would improve readability, enhance logical organization, and make it easier for readers to follow the different analytical approaches used in the study.

RESULTS

240-245: In its current form, this paragraph reads more like an interpretative summary appropriate for the Discussion section rather than a presentation of results. If it is to remain in the Results section, I suggest supplementing it with relevant quantitative data and/or statistical results to support the statements made.

247-253: Consider providing quantitative data on Mesostigmata density rather than relying solely on qualitative descriptions such as “markedly higher” or “lower densities”. Including actual density values (and relevant statistical results) would allow readers to better evaluate the magnitude of the observed differences between treatments and soil types. This also applies to the remainder of the Results section.

294: It would be advisable to include taxonomic authority information when a species is mentioned for the first time in the manuscript. In accordance with zoological nomenclature conventions, the species name should be accompanied by the author citation (authority) upon its first occurrence, e.g. *Tectocepheus velatus sarekensis* Trägårdh.

306: Same comment applies;

DISCUSSION

373: It may be worth supporting this statement with an appropriate citation(s) demonstrating active dispersal as a key mechanism of mite community recovery and its relative importance compared to other processes.

382: Please ensure the citation follows the correct formatting style, and consider adding additional references supporting this type of impact, particularly regarding recovery mechanisms such as active dispersal and population recolonisation dynamics.

393-394: Please add an appropriate citation to support this statement.

397-398: Same comment applies; please add a supporting citation.

420: Please ensure the citation follows the correct formatting style.

434: The species name should be accompanied by the author citation.

521: As above

CONCLUSIONS: The conclusion section is rather lengthy and overlaps with discussion-level detail. I recommend condensing it to a more concise summary of the main findings and implications, without repeating specific taxon-level results or mechanistic explanations.

REFERENCES:

The authors should carefully recheck the entire reference section for consistency and formatting accuracy. (for example, surnames are written in uppercase letters)

SUPPLEMENTARY

Please consider whether S4 Fig. (“Spearman correlations between mite group densities (log(x + 1)) and the most important soil environmental variables across the two soil types”) should be included in the main manuscript rather than as supplementary material, as it presents relatively important data.
