# Supplementary material for: Fine-scale recovery of mite communities following local extinctions on grasslands
Source: PLoS One. 2026 Sep 1;21(9):e0357403. doi: 10.1371/journal.pone.0357403 (PMC13533364; doi:10.1371/journal.pone.0357403)
Supplement: Supplementary file 11 [file pone.0357403.s011.docx]

**Response to the Editor:**

1. Please ensure that your manuscript meets PLOS ONE's style requirements, including those for file naming. The PLOS ONE style templates can be found at

https://journals.plos.org/plosone/s/file?id=wjVg/PLOSOne_formatting_sample_main_body.pdf and

<https://journals.plos.org/plosone/s/file?id=ba62/PLOSOne_formatting_sample_title_authors_affiliations.pdf>

**R: We checked the style of the manuscript.**

2. In your Methods section, please provide additional information regarding the permits you obtained for the work. Please ensure you have included the full name of the authority that approved the field site access and, if no permits were required, a brief statement explaining why.

**R: We added a statement into the Methods section in line (marked version): 147-148.**

3. Please note that PLOS One has specific guidelines on code sharing for submissions in which author-generated code underpins the findings in the manuscript. In these cases, we expect all author-generated code to be made available without restrictions upon publication of the work. Please review our guidelines at https://journals.plos.org/plosone/s/materials-and-software-sharing#loc-sharing-code and ensure that your code is shared in a way that follows best practice and facilitates reproducibility and reuse."

**R: We believe that the R code does not form the central to the manuscript, as we used commands from built-in packages with the parameters described in the methods section. Where a more detailed description of the model structure (SEM) was required, we have set out the structure of the individual models in the supplemental material.**

4. Thank you for stating the following financial disclosure:

“NKFIH Postdoctoral Scholarship (FK 146337)

European Regional Development Fund, and Hungarian Government (GINOP-2.3.2-15-2016-00056)”

Please state what role the funders took in the study. If the funders had no role, please state: "The funders had no role in study design, data collection and analysis, decision to publish, or preparation of the manuscript."

If this statement is not correct you must amend it as needed.

Please include this amended Role of Funder statement in your cover letter; we will change the online submission form on your behalf.

**R: We included this statement in the cover letter above.**

5. Please note that your Data Availability Statement is currently missing the repository name and/or the DOI/accession number of each dataset OR a direct link to access each database. If your manuscript is accepted for publication, you will be asked to provide these details on a very short timeline. We therefore suggest that you provide this information now, though we will not hold up the peer review process if you are unable.

**R: We will make up for it.**
[**https://zenodo.org/records/20084648**](https://zenodo.org/records/20084648)

6. Please upload a new copy of Figure 2 and 4 as the detail is not clear. Please follow the link for more information: https://journals.plos.org/plosone/s/figures.

**R: We uploaded a new version of these figures.**

7. We note you have included a table to which you do not refer in the text of your manuscript. Please ensure that you refer to Table 1 in your text; if accepted, production will need this reference to link the reader to the Table.

**R: We do not have Table 1, so we deleted the word “Table 1” from the main text.**

8. If the reviewer comments include a recommendation to cite specific previously published works, please review and evaluate these publications to determine whether they are relevant and should be cited. There is no requirement to cite these works unless the editor has indicated otherwise.

**R: The reviewer did not recommend to cite specific published works.**

Additional Editor Comments:

The reviewers have provided highly appropriate recommendations, which I ask you to follow for acceptance of the paper. Additionally, it is essential that you state the number of replicates of your experimental treatments and consider carefully if the number of observations is sufficient for your SEM analyses.

**R: We acknowledge the editor’s concern regarding sample size. While n = 144 is modest for a complex SEM, the adequacy of the sample is best evaluated through model fit indices rather than by rules of thumb alone. Both goodness-of-fit tests yielded non-significant p-values (e.g. Fisher's C = 55.845, p = 0.405; χ² = 34.332, p = 0.19), indicating that the model structure is well-supported by the data. Furthermore, the stability of parameter estimates and the moderate-to-high R² values for most response variables suggest that the sample size was sufficient for the present analysis. We nonetheless acknowledge this as a limitation and recommend that future studies replicate these findings with larger sample sizes. We added some new sentences to confirm these limitations in lines: 279-285.**

**--------------------------------------------------------------------------------------------------------------------------------------**

**Responses to Reviewers**

**Reviewer #1:** These findings provide valuable insights for researchers studying soil fauna in disturbed agricultural systems, particularly in understanding taxon-specific recovery dynamics and the role of environmental context in shaping resilience. However, the manuscript requires some revisions prior to publication, which are outlined below.

**R: We sincerely thank you for your constructive comments and valuable suggestions. We have carefully revised the manuscript to address all of the points raised and believe that these changes have substantially improved its quality. Below, we provide a detailed, point-by-point response to each comment. For clarity, we indicate the corresponding line numbers based on the marked version of the revised manuscript.**

Abstract:
It is recommended to shorten the abstract, particularly by simplifying some of the long sentences, which would improve its clarity and enhance readability for a broader audience. In its current form, the abstract is somewhat information-dense, which may make it more difficult to quickly grasp the main results of the study.

**R: Thank you for your comment. We have improved the readability of the Abstract by dividing the longest sentences into shorter ones (lines 29-32), thereby enhancing its clarity. In addition, we deleted the results of Prostigmata from the Abstract. Although another reviewer recommended including additional information, which resulted some more lines and a, we believe, clearer and more informative summary.**

Introduction
Lines 88-99: The paragraph addressing disturbances to soil fauna communities largely repeats concepts that have already been introduced earlier in the introduction, particularly regarding the effects of agricultural practices, spatial patchiness, and biodiversity loss. To improve the overall clarity and conciseness of the manuscript, this section could be merged with the preceding discussion and more tightly integrated. A more compact formulation would help avoid redundancy and strengthen the logical flow of the argument.

**R: Thank you for your comment. We have revised this section to improve clarity and readability by making the paragraphs more concise. In particular, we merged several paragraphs and ideas to create a clearer and more coherent line of reasoning (lines 81-124).**

Additionally, the concepts of resilience and recovery are repeated in several parts of the introduction, leading to redundancy. It is recommended to consolidate this idea into a single, more concise section to improve clarity and coherence of the text.

**R: Thank you for this comment. You are correct that the term “resilience” was not used clearly in the original version. We have revised the manuscript to clarify and use this term more consistently and precisely (lines 122-124).**

Lines 110-115: The hypotheses are clearly formulated and relevant to the study objectives. However, the authors may consider strengthening this section by supporting the individual hypotheses with appropriate citations from the existing literature. This would help further contextualize the predictions and better justify their theoretical basis.

**R: Thank you for your comment. We added some references to the hypotheses; lines: 134, 136, 138.**

Materials and methods

Lines 118-124: The authors should consider adding an appropriate citation for the WRB soil classification to ensure proper referencing of the soil taxonomy used. In addition, the citation format (Gergócs et al., 2022) should be revised to ensure consistency with the citation style used throughout the manuscript.

**R: Thank you for your comment. We have added the appropriate citation for the soil types (line 144) and corrected the formatting of the citation (line 149) to ensure consistency with the journal style.**

Lines 159-160: The list of plant species would benefit from standardisation by adding the taxonomic authority to the Latin names (e.g. Festuca rubra L.). This would ensure consistency with botanical nomenclature conventions and improve taxonomic precision throughout the manuscript.

**R: Thank you for this comment, and we apologize for the oversight. We have corrected the issue in the manuscript also adding the years (lines 184-186).**

Lines 177-198: The Methods section would benefit from clearer structuring. In particular, it is recommended to separate the methodological description into distinct subsections for biological analyses and chemical/physicochemical analyses. This would improve readability, enhance logical organization, and make it easier for readers to follow the different analytical approaches used in the study.

**R: We restructured this section separating the biological and chemical samplings and analyses in lines: 202-235.**

RESULTS
240-245: In its current form, this paragraph reads more like an interpretative summary appropriate for the Discussion section rather than a presentation of results. If it is to remain in the Results section, I suggest supplementing it with relevant quantitative data and/or statistical results to support the statements made.

**R: Thank you for this suggestion. We have removed this section from the manuscript (lines: 288-293).**

247-253: Consider providing quantitative data on Mesostigmata density rather than relying solely on qualitative descriptions such as “markedly higher” or “lower densities”. Including actual density values (and relevant statistical results) would allow readers to better evaluate the magnitude of the observed differences between treatments and soil types. This also applies to the remainder of the Results section.

**R: Thank you for this valuable suggestion. We agree that it improves the clarity of the presentation. We had not implemented this approach initially due to difficulties in determining the most appropriate combination of metrics (effect size vs. density) across the different factor levels. However, we have now resolved this issue and revised the Results section accordingly. We believe that the results are now presented in a clearer and more accessible way throughout the Results section (in lines: 296-298, 300, 305, 308, 316, 318, 325, 331-334, 337-340, 354-355, 358, 360, 362-364, 392-402, 411-419, 425-427).**

294: It would be advisable to include taxonomic authority information when a species is mentioned for the first time in the manuscript. In accordance with zoological nomenclature conventions, the species name should be accompanied by the author citation (authority) upon its first occurrence, e.g. Tectocepheus velatus sarekensis Trägårdh.
306: Same comment applies;

**R: Thank you for the comment. We corrected this mistake in the following lines: 370, 374.**

DISCUSSION
373: It may be worth supporting this statement with an appropriate citation(s) demonstrating active dispersal as a key mechanism of mite community recovery and its relative importance compared to other processes.

**R: Thank you for this suggestion. The original sentence was intended as a forward reference to information provided later in the manuscript. However, we agree that its formulation in the declarative mood could lead to misinterpretation. We have therefore revised the sentence to improve clarity and avoid ambiguity. Line: 467.**

382: Please ensure the citation follows the correct formatting style, and consider adding additional references supporting this type of impact, particularly regarding recovery mechanisms such as active dispersal and population recolonisation dynamics.

**R: Thank you for this comment. We have corrected the citation formatting (lines 477-478) and added two supporting references to strengthen this statement. Minor and Cianciolo (2007) report that Mesostigmata communities may show similar composition in disturbed and undisturbed plots due to their population dynamics and high local mobility. Similarly, Lindberg et al. (2006) found that Mesostigmata recovered rapidly after severe drought, which they attributed to their mobility and ability to undertake horizontal migrations in search of suitable microhabitats (line 478).**

393-394: Please add an appropriate citation to support this statement.

**R: Thank you for this comment. We added the work of Janne Bengtsson (2002) titled: Disturbance and resilience in soil animal communities. Line: 492.**

397-398: Same comment applies; please add a supporting citation.

**R: Thank you for this comment, and for drawing our attention to this point. We have added the relevant citations to support the statement (line 496).**

420: Please ensure the citation follows the correct formatting style.

**R: We corrected this mistake. Line 524.**

434: The species name should be accompanied by the author citation.

**R: Thank you, we corrected it. Line: 543.**

521: As above

**R: Thank you, we corrected it. Line: 625.**

CONCLUSIONS: The conclusion section is rather lengthy and overlaps with discussion-level detail. I recommend condensing it to a more concise summary of the main findings and implications, without repeating specific taxon-level results or mechanistic explanations.

**R: Thank you for this suggestion. We have revised the Conclusion section accordingly, focusing on the main findings and broader implications, and removing taxon-specific details (lines 669-677).**

REFERENCES:
The authors should carefully recheck the entire reference section for consistency and formatting accuracy. (for example, surnames are written in uppercase letters)

**R: We corrected the uppercase names in the references.**

SUPPLEMENTARY
Please consider whether S4 Fig. (“Spearman correlations between mite group densities (log(x + 1)) and the most important soil environmental variables across the two soil types”) should be included in the main manuscript rather than as supplementary material, as it presents relatively important data.

**R: Thank you for this suggestion. We have moved Fig. S4 into the main text, where it is now presented as Fig. 5. As a result, the structural equation model figures have been renumbered as Figs. 6–7.**

**----------------------------------------------------------------------------------------------------------------------------------**

**Reviewer #2**: The manuscript by Gergócs-Winkler and Flórián is devoted to the recovery of soil mite communities in managed grasslands. Overall, the paper is very nicely written and concerns important and novel research questions. Few comments below will help to improve the paper further. The submission may be considered for publication after a major revision.

**Response: We sincerely thank you for your constructive comments and valuable suggestions. We have carefully revised the manuscript to address all of the points raised and believe that these changes have substantially improved its quality. Below, we provide a detailed, point-by-point response to each comment. For clarity, we indicate the corresponding line numbers based on the marked version of the revised manuscript.**

General comments
I am not that familiar with the journal formatting rules, but adding page numbers would help.

**R: Thank you for the comment, we added page numbers to the manuscript.**

The abstract is a bit too general. Enriching it with specific recovery rates would make it more insightful.

**R: Thank you for your comment. We have added the mean effect size values to the Abstract explaining “specific recovery rates”, as this metric is exact and is used consistently throughout the manuscript. However, this was not straightforward, as results are reported for multiple taxonomic groups across three years, two soil types, and two seasons. Consequently, we present overall mean values in the Abstract, which necessarily provide a simplified summary and may not fully capture all temporal and soil-specific patterns observed in the results. However, if we were to include all these considerations, it would make the abstract very complicated. (Lines: 18-20, 26)**

I am not sure, that direct comparison of the two sites makes sense as at this level data is pseudoreplicated. One would need at least two locations per soil type then. Despite soil types provided highly contrasting sets of abiotic conditions, still, some true replication would be needed to avoid false positive results.

**R: Thank you for this comment. We agree with this point. Unfortunately, it is not possible to repeat the study at both sites at this time. However, we have now explicitly acknowledged this limitation in the manuscript (lines 616-618).**

Specific comments

Abstract
L12 “to respond sensitively to environmental change” this phrase is too unspecific. You probably mean community level response or something.

**R: Thank you for this comment. You are correct that the original sentence was too unspecific. We have revised it accordingly (lines 11-12).**

L13 you are mentioning agricultural fields here, which subconsciously are recognized as arable fields. Since you are talking about grasslands (meadows) it might be useful to use consistent terminology that would exclude any dubious statements and terms.

**R: Thank you for this comment. We agree that this point may have been confusing. The experiment was conducted in an area that was formerly an agricultural crop field, but at the time of the study it had been newly established as a grassland. We therefore corrected the description (line 13).**

L23 “In contrast, oribatid mites”

**R: Thank you, we corrected it in line 25.**

L29 I think, it would be safer here to talk about recovery assessments, as resilience includes recovery share derived from migration.

**R: Thank you for your comment. We have corrected the term to “recovery”. In addition, the sentence was slightly modified in response to other reviewers’ suggestions (line 32).**

Introduction:
L38 “plays”

**R: Thank you for this comment. We would like to clarify that “biota” is a collective noun referring to living organisms as a whole and is therefore treated as plural in scientific usage. Accordingly, the correct form is “biota play,” and we have retained this usage in the revised manuscript (line 43).**

L103 Overall, if introduced, the authors need to explain, which understanding of resilience is meant here. Are you operating with resilience sensu Pimm or some other? Maybe, it would be a safer strategy to avoid operating with resilience at all.

**R: Thank you for this important comment. We have clarified our definition of resilience in the manuscript to make our usage of the term more explicit and consistent (lines 122-124).**

Materials and Methods
L118 “with two different soil types”

**R: Thank you, we corrected it: line 141.**

L148 “pore size”

**R: We corrected it. Line 173.**

L168 “cylindrical”

**R: We corrected this word. Line 204.**

L169 Please, rephrase, reads awkwardly,

**R: Thank you, we rephrased this part. Lines 205-206.**

L179-188 Though the intent of the authors to identify material to the finest possible level is appreciated, such multi-resolution approach complicates understanding of recovery patterns as we can talk rather about density recovery per higher taxa, but not making a true community level analysis. There is seemingly not much can be done with that, but at least some discussion on the limitations of such approach would be helpful.

**R: Thank you for this comment. We have added a paragraph highlighting this limitation in the manuscript (lines 237-240).**

L200 Please, mention R-version here as well.

**R: We added this information, line: 242.**

Discussion:
L361-370 I do not believe, this is needed.

**R: Thank you for this simplifying suggestion, we deleted this section (lines 454-463).**

L371 The subtitle is highly uninformative.

**R: We deleted the subtitles (lines 464, 567).**

L379-381 and elsewhere: please, avoid such extensive results repetitions. Proceed directly to interpretations.

**R: Thank you for this helpful suggestion. We have rewrite several of these sentences (see lines 473, 485, 504, 517, 520, 529, 535, 548, 569, 583).**

L498 as said below – direct comparisons of recovery patterns in the two soil types is risky due to the lack of true replication.

**R: Thank you for pointing this out. We agree and have added a section in the manuscript explicitly addressing this limitation (lines 616-618).**

L509 and elsewhere: use “sexually reproducing species” instead.

**R: Thank you for the suggestion. We corrected this term in the manuscript: Lines: 79, 381, 383, 387, 630, 631, 635.**
