# Supplementary material for: Fine-scale recovery of mite communities following local extinctions on grasslands
Source: PLoS One. 2026 Sep 1;21(9):e0357403. doi: 10.1371/journal.pone.0357403 (PMC13533364; doi:10.1371/journal.pone.0357403)
Supplement: Supplementary file 12 [file pone.0357403.s012.docx]

**Response (R) to the Editor:**

Thank you very much for your thorough editing, which has helped improve the quality of the manuscript. Below, we provide our responses to your comments. The line numbers refer to the **unmarked** version of the revised manuscript.

L. 140: Please replace “dried in” to “dried at”.

R: Thank you for your comment. We have corrected this as suggested (L. 140).

L. 155-157: Under the International Code of Nomenclature for algae, fungi, and plants (<https://www.iapt-taxon.org/nomen/main.php>), plant scientific names conventionally include a standard author abbrevia-tion, but omit the year of publication. Authority should not be italics, and authority should only be in pa-rentheses in cases where the original name of the species has changed. Please check and correct.

R: Thank you for your comment and for the detailed instructions. We apologise for not having checked these rules carefully enough. We have now corrected all the plant scientific names accordingly (L. 155-156).

L. 321, 325, 334, 552: Scientific names: Authority and year should not be in italics and only in parentheses in cases where the original name of the species has changed. Please check and correct, see International Commission on Zoological Nomenclature (<https://www.iczn.org/outreach/faqs/>).

R: Thank you for your comment and for the detailed instructions. We apologise for not having checked these rules carefully enough. We have now corrected all the scientific names accordingly (L. 324, 328, 337, 554).

L. 342 and elsewhere: What does the unit “m/m%” denote? Please use an SI unit.

R: Thank you for your comment. We agree that m/m% is not an SI unit and have therefore converted these values to g/kg (L. 345–348). We have also corrected the units in the Supplementary Material. As a result of this conversion, the numerical values are ten times higher.

L. 473, 532: spp. should not be in italics.

R: Thank you for pointing this out. We have corrected the formatting of spp. accordingly (L. 475, 534).

**Response to Reviewer 2:**

Thank you for carefully addressing all comments. I have one minor addition though: currently figure 1 caption looks incomplete. Please, review and revise.

R: Thank you for your comment. We have revised and expanded the caption of Figure 1 to provide the additional information needed to clarify the figure (L. 182-187).
