# Supplementary material for: Fine-scale recovery of mite communities following local extinctions on grasslands
Source: PLoS One. 2026 Sep 1;21(9):e0357403. doi: 10.1371/journal.pone.0357403 (PMC13533364; doi:10.1371/journal.pone.0357403)
Supplement: Supplementary file 13 [file pone.0357403.s001.docx]

**S1 Fig. Photos of the experimental design and the interior of the mesocosms with grass plants.**
