# Supplementary material for: Fine-scale recovery of mite communities following local extinctions on grasslands
Source: PLoS One. 2026 Sep 1;21(9):e0357403. doi: 10.1371/journal.pone.0357403 (PMC13533364; doi:10.1371/journal.pone.0357403)
Supplement: Peer review history

# Peer review: Decision Letter 0

**DOI:** 10.1371/journal.pone.0357403.r001

**Authors:** Mette Vestergård

18 Jun 2026

Dear Dr. Veronika,

Thank you for submitting your manuscript to PLOS ONE. After careful consideration, we feel that it has merit but does not fully meet PLOS ONE’s publication criteria as it currently stands. Therefore, we invite you to submit a revised version of the manuscript that addresses the points raised during the review process.

Please submit your revised manuscript by Aug 17 2026 11:59PM. If you will need more time than this to complete your revisions, please reply to this message or contact the journal office at plosone@plos.org. When you're ready to submit your revision, log on to https://www.editorialmanager.com/pone/ and select the 'Submissions Needing Revision' folder to locate your manuscript file.

- A letter that responds to each point raised by the academic editor and reviewer(s). You should upload this letter as a separate file labeled 'Response to Reviewers'.
- A marked-up copy of your manuscript that highlights changes made to the original version. You should upload this as a separate file labeled 'Revised Manuscript with Track Changes'.
- An unmarked version of your revised paper without tracked changes. You should upload this as a separate file labeled 'Manuscript'.

If you would like to make changes to your financial disclosure, please include your updated statement in your cover letter. Guidelines for resubmitting your figure files are available below the reviewer comments at the end of this letter.

If applicable, we recommend that you deposit your laboratory protocols in protocols.io to enhance the reproducibility of your results. Protocols.io assigns your protocol its own identifier (DOI) so that it can be cited independently in the future. For instructions see: https://journals.plos.org/plosone/s/submission-guidelines#loc-laboratory-protocols. Additionally, PLOS ONE offers an option for publishing peer-reviewed Lab Protocol articles, which describe protocols hosted on protocols.io. Read more information on sharing protocols at https://plos.org/protocols?utm_medium=editorial-email&utm_source=authorletters&utm_campaign=protocols.

As the corresponding author, your ORCID iD is verified in the submission system and will appear in the published article. PLOS supports the use of ORCID, and we encourage all coauthors to register for an ORCID iD and use it as well. Please encourage your coauthors to verify their ORCID iD within the submission system before final acceptance, as unverified ORCID iDs will not appear in the published article. *Only*  the individual author can complete the verification step; PLOS staff *cannot*  verify ORCID iDs on behalf of authors.

We look forward to receiving your revised manuscript.

Kind regards,

Mette Vestergård, Ph.D.

Academic Editor

PLOS One

Journal Requirements:

When submitting your revision, we need you to address these additional requirements.

1.Please ensure that your manuscript meets PLOS ONE's style requirements, including those for file naming. The PLOS ONE style templates can be found at

https://journals.plos.org/plosone/s/file?id=wjVg/PLOSOne_formatting_sample_main_body.pdf and

https://journals.plos.org/plosone/s/file?id=ba62/PLOSOne_formatting_sample_title_authors_affiliations.pdf

2. In your Methods section, please provide additional information regarding the permits you obtained for the work. Please ensure you have included the full name of the authority that approved the field site access and, if no permits were required, a brief statement explaining why.

3. Please note that PLOS One has specific guidelines on code sharing for submissions in which author-generated code underpins the findings in the manuscript. In these cases, we expect all author-generated code to be made available without restrictions upon publication of the work. Please review our guidelines at https://journals.plos.org/plosone/s/materials-and-software-sharing#loc-sharing-code and ensure that your code is shared in a way that follows best practice and facilitates reproducibility and reuse."

4. Thank you for stating the following financial disclosure:

“NKFIH Postdoctoral Scholarship (FK 146337)

European Regional Development Fund, and Hungarian Government (GINOP-2.3.2-15-2016-00056)”

Please state what role the funders took in the study.  If the funders had no role, please state: "The funders had no role in study design, data collection and analysis, decision to publish, or preparation of the manuscript."

If this statement is not correct you must amend it as needed.

Please include this amended Role of Funder statement in your cover letter; we will change the online submission form on your behalf.

5. Please note that your Data Availability Statement is currently missing the repository name and/or the DOI/accession number of each dataset OR a direct link to access each database. If your manuscript is accepted for publication, you will be asked to provide these details on a very short timeline. We therefore suggest that you provide this information now, though we will not hold up the peer review process if you are unable.

6. Please upload a new copy of Figure 2 and 4 as the detail is not clear. Please follow the link for more information:  https://journals.plos.org/plosone/s/figures.

7. We note you have included a table to which you do not refer in the text of your manuscript. Please ensure that you refer to Table 1 in your text; if accepted, production will need this reference to link the reader to the Table.

8. If the reviewer comments include a recommendation to cite specific previously published works, please review and evaluate these publications to determine whether they are relevant and should be cited. There is no requirement to cite these works unless the editor has indicated otherwise.

Additional Editor Comments:

The reviewers have provided highly appropriate recommendations, which I ask you to follow for acceptance of the paper. Additionally, it is essential that you state the number of replicates of your experimental treatments and consider carefully if the number of observations is sufficient for your SEM analyses.

[Note: HTML markup is below. Please do not edit.]

Reviewer's Responses to Questions

**Comments to the Author**

1. Is the manuscript technically sound, and do the data support the conclusions?

Reviewer #1: Yes

Reviewer #2: Yes

**********

2. Has the statistical analysis been performed appropriately and rigorously?

Reviewer #1: Yes

Reviewer #2: Yes

**********

3. Have the authors made all data underlying the findings in their manuscript fully available?

The PLOS Data policy

Reviewer #1: Yes

Reviewer #2: Yes

**********

4. Is the manuscript presented in an intelligible fashion and written in standard English?

Reviewer #1: Yes

Reviewer #2: Yes

**********

Reviewer #1: These findings provide valuable insights for researchers studying soil fauna in disturbed agricultural systems, particularly in understanding taxon-specific recovery dynamics and the role of environmental context in shaping resilience. However, the manuscript requires some revisions prior to publication, which are outlined below.

Abstract:

It is recommended to shorten the abstract, particularly by simplifying some of the long sentences, which would improve its clarity and enhance readability for a broader audience. In its current form, the abstract is somewhat information-dense, which may make it more difficult to quickly grasp the main results of the study.

Intorduction:

Lines 88-99: The paragraph addressing disturbances to soil fauna communities largely repeats concepts that have already been introduced earlier in the introduction, particularly regarding the effects of agricultural practices, spatial patchiness, and biodiversity loss. To improve the overall clarity and conciseness of the manuscript, this section could be merged with the preceding discussion and more tightly integrated. A more compact formulation would help avoid redundancy and strengthen the logical flow of the argument.

Additionally, the concepts of resilience and recovery are repeated in several parts of the introduction, leading to redundancy. It is recommended to consolidate this idea into a single, more concise section to improve clarity and coherence of the text.

Lines 110-115: The hypotheses are clearly formulated and relevant to the study objectives. However, the authors may consider strengthening this section by supporting the individual hypotheses with appropriate citations from the existing literature. This would help further contextualize the predictions and better justify their theoretical basis.

Materials and methods

Lines 118-124: The authors should consider adding an appropriate citation for the WRB soil classification to ensure proper referencing of the soil taxonomy used. In addition, the citation format (Gergócs et al., 2022) should be revised to ensure consistency with the citation style used throughout the manuscript.

Lines 159-160: The list of plant species would benefit from standardisation by adding the taxonomic authority to the Latin names (e.g. Festuca rubra L.). This would ensure consistency with botanical nomenclature conventions and improve taxonomic precision throughout the manuscript.

Lines 177-198: The Methods section would benefit from clearer structuring. In particular, it is recommended to separate the methodological description into distinct subsections for biological analyses and chemical/physicochemical analyses. This would improve readability, enhance logical organization, and make it easier for readers to follow the different analytical approaches used in the study.

RESULTS

240-245: In its current form, this paragraph reads more like an interpretative summary appropriate for the Discussion section rather than a presentation of results. If it is to remain in the Results section, I suggest supplementing it with relevant quantitative data and/or statistical results to support the statements made.

247-253: Consider providing quantitative data on Mesostigmata density rather than relying solely on qualitative descriptions such as “markedly higher” or “lower densities”. Including actual density values (and relevant statistical results) would allow readers to better evaluate the magnitude of the observed differences between treatments and soil types. This also applies to the remainder of the Results section.

294: It would be advisable to include taxonomic authority information when a species is mentioned for the first time in the manuscript. In accordance with zoological nomenclature conventions, the species name should be accompanied by the author citation (authority) upon its first occurrence, e.g. Tectocepheus velatus sarekensis Trägårdh.

306: Same comment applies;

DISCUSSION

373: It may be worth supporting this statement with an appropriate citation(s) demonstrating active dispersal as a key mechanism of mite community recovery and its relative importance compared to other processes.

382: Please ensure the citation follows the correct formatting style, and consider adding additional references supporting this type of impact, particularly regarding recovery mechanisms such as active dispersal and population recolonisation dynamics.

393-394: Please add an appropriate citation to support this statement.

397-398: Same comment applies; please add a supporting citation.

420: Please ensure the citation follows the correct formatting style.

434: The species name should be accompanied by the author citation.

521: As above

CONCLUSIONS: The conclusion section is rather lengthy and overlaps with discussion-level detail. I recommend condensing it to a more concise summary of the main findings and implications, without repeating specific taxon-level results or mechanistic explanations.

REFERENCES:

The authors should carefully recheck the entire reference section for consistency and formatting accuracy. (for example, surnames are written in uppercase letters)

SUPPLEMENTARY

Please consider whether S4 Fig. (“Spearman correlations between mite group densities (log(x + 1)) and the most important soil environmental variables across the two soil types”) should be included in the main manuscript rather than as supplementary material, as it presents relatively important data.

Reviewer #2: The manuscript by Gercocs and Florian is devoted to the recovery of soil mite communities in managed grasslands. Overall, the paper is very nicely written and concerns important and novel research questions. Few comments below will help to improve the paper further. The submission may be considered for publication after a major revision.

General comments

I am not that familiar with the journal formatting rules, but adding page numbers would help.

The abstract is a bit too general. Enriching it with specific recovery rates would make it more insightful.

I am not sure, that direct comparison of the two sites makes sense as at this level data is pseudoreplicated. One would need at least two locations per soil type then. Despite soil types provided highly contrasting sets of abiotic conditions, still, some true replication would be needed to avoid false positive results.

Specific comments

L12 “to respond sensitively to environmental change” this phrase is too unspecific. You probably mean community level response or something.

L13 you are mentioning agricultural fields here, which subconsciously are recognized as arable fields. Since you are talking about grasslands (meadows) it might be useful to use consistent terminology that would exclude any dubious statements and terms.

L23 “In contrast, oribatid mites”

L29 I think, it would be safer here to talk about recovery assessments, as resilience includes recovery share derived from migration.

L38 “plays”

L103 Overall, if introduced, the authors need to explain, which understanding of resilience is meant here. Are you operating with resilience sensu Pimm or some other? Maybe, it would be a safer strategy to avoid operating with resilience at all.

L118 “with two different soil types”

L148 “pore size”

L168 “cylindrical”

L169 Please, rephrase, reads awkwardly,

L179-188 Though the intent of the authors to identify material to the finest possible level is appreciated, such multi-resolution approach complicates understanding of recovery patterns as we can talk rather about density recovery per higher taxa, but not making a true community level analysis. There is seemingly not much can be done with that, but at least some discussion on the limitations of such approach would be helpful.

L200 Please, mention R-version here as well.

L361-370 I do not believe, this is needed.

L371 The subtitle is highly uninformative.

L379-381 and elsewhere: please, avoid such extensive results repetitions. Proceed directly to interpretations.

L498 as said below – direct comparisons of recovery patterns in the two soil types is risky due to the lack of true replication.

L509 and elsewhere: use “sexually reproducing species” instead.

**********

what does this mean?). If published, this will include your full peer review and any attached files.

If you choose “no”, your identity will remain anonymous but your review may still be made public.

**Do you want your identity to be public for this peer review?**  For information about this choice, including consent withdrawal, please see our Privacy Policy

Reviewer #1: No

Reviewer #2: No

**********

[NOTE: If reviewer comments were submitted as an attachment file, they will be attached to this email and accessible via the submission site. Please log into your account, locate the manuscript record, and check for the action link "View Attachments". If this link does not appear, there are no attachment files.]

To ensure your figures meet our technical requirements, please review our figure guidelines: https://journals.plos.org/plosone/s/figures

You may also use PLOS’s free figure tool, NAAS, to help you prepare publication quality figures: https://journals.plos.org/plosone/s/figures#loc-tools-for-figure-preparation.

NAAS will assess whether your figures meet our technical requirements by comparing each figure against our figure specifications.

Attachment Submitted filename: Review 09.06.docx

# Author comment: Author response to Decision Letter 1

**DOI:** 10.1371/journal.pone.0357403.r002

28 Jul 2026

Response to the Editor:

1. Please ensure that your manuscript meets PLOS ONE's style requirements, including those for file naming. The PLOS ONE style templates can be found at

https://journals.plos.org/plosone/s/file?id=wjVg/PLOSOne_formatting_sample_main_body.pdf and

https://journals.plos.org/plosone/s/file?id=ba62/PLOSOne_formatting_sample_title_authors_affiliations.pdf

R: We checked the style of the manuscript.

2. In your Methods section, please provide additional information regarding the permits you obtained for the work. Please ensure you have included the full name of the authority that approved the field site access and, if no permits were required, a brief statement explaining why.

R: We added a statement into the Methods section in line (marked version): 147-148.

3. Please note that PLOS One has specific guidelines on code sharing for submissions in which author-generated code underpins the findings in the manuscript. In these cases, we expect all author-generated code to be made available without restrictions upon publication of the work. Please review our guidelines at https://journals.plos.org/plosone/s/materials-and-software-sharing#loc-sharing-code and ensure that your code is shared in a way that follows best practice and facilitates reproducibility and reuse."

R: We believe that the R code does not form the central to the manuscript, as we used commands from built-in packages with the parameters described in the methods section. Where a more detailed description of the model structure (SEM) was required, we have set out the structure of the individual models in the supplemental material.

4. Thank you for stating the following financial disclosure:

“NKFIH Postdoctoral Scholarship (FK 146337)

European Regional Development Fund, and Hungarian Government (GINOP-2.3.2-15-2016-00056)”

Please state what role the funders took in the study. If the funders had no role, please state: "The funders had no role in study design, data collection and analysis, decision to publish, or preparation of the manuscript."

If this statement is not correct you must amend it as needed.

Please include this amended Role of Funder statement in your cover letter; we will change the online submission form on your behalf.

R: We included this statement in the cover letter above.

5. Please note that your Data Availability Statement is currently missing the repository name and/or the DOI/accession number of each dataset OR a direct link to access each database. If your manuscript is accepted for publication, you will be asked to provide these details on a very short timeline. We therefore suggest that you provide this information now, though we will not hold up the peer review process if you are unable.

R: We will make up for it.

https://zenodo.org/records/20084648

6. Please upload a new copy of Figure 2 and 4 as the detail is not clear. Please follow the link for more information: https://journals.plos.org/plosone/s/figures.

R: We uploaded a new version of these figures.

7. We note you have included a table to which you do not refer in the text of your manuscript. Please ensure that you refer to Table 1 in your text; if accepted, production will need this reference to link the reader to the Table.

R: We do not have Table 1, so we deleted the word “Table 1” from the main text.

8. If the reviewer comments include a recommendation to cite specific previously published works, please review and evaluate these publications to determine whether they are relevant and should be cited. There is no requirement to cite these works unless the editor has indicated otherwise.

R: The reviewer did not recommend to cite specific published works.

Additional Editor Comments:

The reviewers have provided highly appropriate recommendations, which I ask you to follow for acceptance of the paper. Additionally, it is essential that you state the number of replicates of your experimental treatments and consider carefully if the number of observations is sufficient for your SEM analyses.

R: We acknowledge the editor’s concern regarding sample size. While n = 144 is modest for a complex SEM, the adequacy of the sample is best evaluated through model fit indices rather than by rules of thumb alone. Both goodness-of-fit tests yielded non-significant p-values (e.g. Fisher's C = 55.845, p = 0.405; χ² = 34.332, p = 0.19), indicating that the model structure is well-supported by the data. Furthermore, the stability of parameter estimates and the moderate-to-high R² values for most response variables suggest that the sample size was sufficient for the present analysis. We nonetheless acknowledge this as a limitation and recommend that future studies replicate these findings with larger sample sizes. We added some new sentences to confirm these limitations in lines: 279-285.

--------------------------------------------------------------------------------------------------------------------------------------

Responses to Reviewers

Reviewer #1: These findings provide valuable insights for researchers studying soil fauna in disturbed agricultural systems, particularly in understanding taxon-specific recovery dynamics and the role of environmental context in shaping resilience. However, the manuscript requires some revisions prior to publication, which are outlined below.

R: We sincerely thank you for your constructive comments and valuable suggestions. We have carefully revised the manuscript to address all of the points raised and believe that these changes have substantially improved its quality. Below, we provide a detailed, point-by-point response to each comment. For clarity, we indicate the corresponding line numbers based on the marked version of the revised manuscript.

Abstract:

It is recommended to shorten the abstract, particularly by simplifying some of the long sentences, which would improve its clarity and enhance readability for a broader audience. In its current form, the abstract is somewhat information-dense, which may make it more difficult to quickly grasp the main results of the study.

R: Thank you for your comment. We have improved the readability of the Abstract by dividing the longest sentences into shorter ones (lines 29-32), thereby enhancing its clarity. In addition, we deleted the results of Prostigmata from the Abstract. Although another reviewer recommended including additional information, which resulted some more lines and a, we believe, clearer and more informative summary.

Introduction

Lines 88-99: The paragraph addressing disturbances to soil fauna communities largely repeats concepts that have already been introduced earlier in the introduction, particularly regarding the effects of agricultural practices, spatial patchiness, and biodiversity loss. To improve the overall clarity and conciseness of the manuscript, this section could be merged with the preceding discussion and more tightly integrated. A more compact formulation would help avoid redundancy and strengthen the logical flow of the argument.

R: Thank you for your comment. We have revised this section to improve clarity and readability by making the paragraphs more concise. In particular, we merged several paragraphs and ideas to create a clearer and more coherent line of reasoning (lines 81-124).

Additionally, the concepts of resilience and recovery are repeated in several parts of the introduction, leading to redundancy. It is recommended to consolidate this idea into a single, more concise section to improve clarity and coherence of the text.

R: Thank you for this comment. You are correct that the term “resilience” was not used clearly in the original version. We have revised the manuscript to clarify and use this term more consistently and precisely (lines 122-124).

Lines 110-115: The hypotheses are clearly formulated and relevant to the study objectives. However, the authors may consider strengthening this section by supporting the individual hypotheses with appropriate citations from the existing literature. This would help further contextualize the predictions and better justify their theoretical basis.

R: Thank you for your comment. We added some references to the hypotheses; lines: 134, 136, 138.

Materials and methods

Lines 118-124: The authors should consider adding an appropriate citation for the WRB soil classification to ensure proper referencing of the soil taxonomy used. In addition, the citation format (Gergócs et al., 2022) should be revised to ensure consistency with the citation style used throughout the manuscript.

R: Thank you for your comment. We have added the appropriate citation for the soil types (line 144) and corrected the formatting of the citation (line 149) to ensure consistency with the journal style.

Lines 159-160: The list of plant species would benefit from standardisation by adding the taxonomic authority to the Latin names (e.g. Festuca rubra L.). This would ensure consistency with botanical nomenclature conventions and improve taxonomic precision throughout the manuscript.

R: Thank you for this comment, and we apologize for the oversight. We have corrected the issue in the manuscript also adding the years (lines 184-186).

Lines 177-198: The Methods section would benefit from clearer structuring. In particular, it is recommended to separate the methodological description into distinct subsections for biological analyses and chemical/physicochemical analyses. This would improve readability, enhance logical organization, and make it easier for readers to follow the different analytical approaches used in the study.

R: We restructured this section separating the biological and chemical samplings and analyses in lines: 202-235.

RESULTS

240-245: In its current form, this paragraph reads more like an interpretative summary appropriate for the Discussion section rather than a presentation of results. If it is to remain in the Results section, I suggest supplementing it with relevant quantitative data and/or statistical results to support the statements made.

R: Thank you for this suggestion. We have removed this section from the manuscript (lines: 288-293).

247-253: Consider providing quantitative data on Mesostigmata density rather than relying solely on qualitative descriptions such as “markedly higher” or “lower densities”. Including actual density values (and relevant statistical results) would allow readers to better evaluate the magnitude of the observed differences between treatments and soil types. This also applies to the remainder of the Results section.

R: Thank you for this valuable suggestion. We agree that it improves the clarity of the presentation. We had not implemented this approach initially due to difficulties in determining the most appropriate combination of metrics (effect size vs. density) across the different factor levels. However, we have now resolved this issue and revised the Results section accordingly. We believe that the results are now presented in a clearer and more accessible way throughout the Results section (in lines: 296-298, 300, 305, 308, 316, 318, 325, 331-334, 337-340, 354-355, 358, 360, 362-364, 392-402, 411-419, 425-427).

294: It would be advisable to include taxonomic authority information when a species is mentioned for the first time in the manuscript. In accordance with zoological nomenclature conventions, the species name should be accompanied by the author citation (authority) upon its first occurrence, e.g. Tectocepheus velatus sarekensis Trägårdh.

306: Same comment applies;

R: Thank you for the comment. We corrected this mistake in the following lines: 370, 374.

DISCUSSION

373: It may be worth supporting this statement with an appropriate citation(s) demonstrating active dispersal as a key mechanism of mite community recovery and its relative importance compared to other processes.

R: Thank you for this suggestion. The original sentence was intended as a forward reference to information provided later in the manuscript. However, we agree that its formulation in the declarative mood could lead to misinterpretation. We have therefore revised the sentence to improve clarity and avoid ambiguity. Line: 467.

382: Please ensure the citation follows the correct formatting style, and consider adding additional references supporting this type of impact, particularly regarding recovery mechanisms such as active dispersal and population recolonisation dynamics.

R: Thank you for this comment. We have corrected the citation formatting (lines 477-478) and added two supporting references to strengthen this statement. Minor and Cianciolo (2007) report that Mesostigmata communities may show similar composition in disturbed and undisturbed plots due to their population dynamics and high local mobility. Similarly, Lindberg et al. (2006) found that Mesostigmata recovered rapidly after severe drought, which they attributed to their mobility and ability to undertake horizontal migrations in search of suitable microhabitats (line 478).

393-394: Please add an appropriate citation to support this statement.

R: Thank you for this comment. We added the work of Janne Bengtsson (2002) titled: Disturbance and resilience in soil animal communities. Line: 492.

397-398: Same comment applies; please add a supporting citation.

R: Thank you for this comment, and for drawing our attention to this point. We have added the relevant citations to support the statement (line 496).

420: Please ensure the citation follows the correct formatting style.

R: We corrected this mistake. Line 524.

434: The species name should be accompanied by the author citation.

R: Thank you, we corrected it. Line: 543.

521: As above

R: Thank you, we corrected it. Line: 625.

CONCLUSIONS: The conclusion section is rather lengthy and overlaps with discussion-level detail. I recommend condensing it to a more concise summary of the main findings and implications, without repeating specific taxon-level results or mechanistic explanations.

R: Thank you for this suggestion. We have revised the Conclusion section accordingly, focusing on the main findings and broader implications, and removing taxon-specific details (lines 669-677).

REFERENCES:

The authors should carefully recheck the entire reference section for consistency and formatting accuracy. (for example, surnames are written in uppercase letters)

R: We corrected the uppercase names in the references.

SUPPLEMENTARY

Please consider whether S4 Fig. (“Spearman correlations between mite group densities (log(x + 1)) and the most important soil environmental variables across the two soil types”) should be included in the main manuscript rather than as supplementary material, as it presents relatively important data.

R: Thank you for this suggestion. We have moved Fig. S4 into the main text, where it is now presented as Fig. 5. As a result, the structural equation model figures have been renumbered as Figs. 6–7.

----------------------------------------------------------------------------------------------------------------------------------

Reviewer #2: The manuscript by Gergócs-Winkler and Flórián is devoted to the recovery of soil mite communities in managed grasslands. Overall, the paper is very nicely written and concerns important and novel research questions. Few comments below will help to improve the paper further. The submission may be considered for publication after a major revision.

Response: We sincerely thank you for your constructive comments and valuable suggestions. We have carefully revised the manuscript to address all of the points raised and believe that these changes have substantially improved its quality. Below, we provide a detailed, point-by-point response to each comment. For clarity, we indicate the corresponding line numbers based on the marked version of the revised manuscript.

General comments

I am not that familiar with the journal formatting rules, but adding page numbers would help.

R: Thank you for the comment, we added page numbers to the manuscript.

The abstract is a bit too general. Enriching it with specific recovery rates would make it more insightful.

R: Thank you for your comment. We have added the mean effect size values to the Abstract explaining “specific recovery rates”, as this metric is exact and is used consistent

Attachment Submitted filename: Response to Reviewers.docx

# Peer review: Decision Letter 1

**DOI:** 10.1371/journal.pone.0357403.r003

**Authors:** Mette Vestergård

5 Aug 2026

Dear Dr. Veronika,

Thank you for submitting your manuscript to PLOS ONE. After careful consideration, we feel that it has merit but does not fully meet PLOS ONE’s publication criteria as it currently stands. Therefore, we invite you to submit a revised version of the manuscript that addresses the points raised during the review process.

==============================

Thanks for submitting your thoroughly revised manuscript. In addition to the comment from Reviewer 2, please correct the following before final acceptance:

L. 140: Please replace “dried in” to “dried at”.

L. 155-157: Under the International Code of Nomenclature for algae, fungi, and plants (https://www.iapt-taxon.org/nomen/main.php), plant scientific names conventionally include a standard author abbrevia-tion, but omit the year of publication. Authority should not be italics, and authority should only be in pa-rentheses in cases where the original name of the species has changed. Please check and correct.

L. 321, 325, 334, 552: Scientific names: Authority and year should not be in italics and only in parentheses in cases where the original name of the species has changed. Please check and correct, see International Commission on Zoological Nomenclature (https://www.iczn.org/outreach/faqs/).

L. 342 and elsewhere: What does the unit “m/m%” denote? Please use an SI unit.

L. 473, 532: spp. should not be in italics.

==============================

Please submit your revised manuscript by Sep 04 2026 11:59PM. If you will need more time than this to complete your revisions, please reply to this message or contact the journal office at plosone@plos.org. When you're ready to submit your revision, log on to https://www.editorialmanager.com/pone/ and select the 'Submissions Needing Revision' folder to locate your manuscript file.

- A letter that responds to each point raised by the academic editor and reviewer(s). You should upload this letter as a separate file labeled 'Response to Reviewers'.
- A marked-up copy of your manuscript that highlights changes made to the original version. You should upload this as a separate file labeled 'Revised Manuscript with Track Changes'.
- An unmarked version of your revised paper without tracked changes. You should upload this as a separate file labeled 'Manuscript'.

If you would like to make changes to your financial disclosure, please include your updated statement in your cover letter. Guidelines for resubmitting your figure files are available below the reviewer comments at the end of this letter.

If applicable, we recommend that you deposit your laboratory protocols in protocols.io to enhance the reproducibility of your results. Protocols.io assigns your protocol its own identifier (DOI) so that it can be cited independently in the future. For instructions see: https://journals.plos.org/plosone/s/submission-guidelines#loc-laboratory-protocols. Additionally, PLOS ONE offers an option for publishing peer-reviewed Lab Protocol articles, which describe protocols hosted on protocols.io. Read more information on sharing protocols at https://plos.org/protocols?utm_medium=editorial-email&utm_source=authorletters&utm_campaign=protocols.

As the corresponding author, your ORCID iD is verified in the submission system and will appear in the published article. PLOS supports the use of ORCID, and we encourage all coauthors to register for an ORCID iD and use it as well. Please encourage your coauthors to verify their ORCID iD within the submission system before final acceptance, as unverified ORCID iDs will not appear in the published article. *Only*  the individual author can complete the verification step; PLOS staff *cannot*  verify ORCID iDs on behalf of authors.

We look forward to receiving your revised manuscript.

Kind regards,

Mette Vestergård, Ph.D.

Academic Editor

PLOS One

Journal Requirements:

If the reviewer comments include a recommendation to cite specific previously published works, please review and evaluate these publications to determine whether they are relevant and should be cited. There is no requirement to cite these works unless the editor has indicated otherwise.

Please review your reference list to ensure that it is complete and correct. If you have cited papers that have been retracted, please include the rationale for doing so in the manuscript text, or remove these references and replace them with relevant current references. Any changes to the reference list should be mentioned in the rebuttal letter that accompanies your revised manuscript. If you need to cite a retracted article, indicate the article’s retracted status in the References list and also include a citation and full reference for the retraction notice.

Additional Editor Comments:

Thanks for submitting your thoroughly revised manuscript. In addition to the comment from Reviewer 2, please correct the following before final acceptance:

L. 140: Please replace “dried in” to “dried at”.

L. 155-157: Under the International Code of Nomenclature for algae, fungi, and plants (https://www.iapt-taxon.org/nomen/main.php), plant scientific names conventionally include a standard author abbrevia-tion, but omit the year of publication. Authority should not be italics, and authority should only be in pa-rentheses in cases where the original name of the species has changed. Please check and correct.

L. 321, 325, 334, 552: Scientific names: Authority and year should not be in italics and only in parentheses in cases where the original name of the species has changed. Please check and correct, see International Commission on Zoological Nomenclature (https://www.iczn.org/outreach/faqs/).

L. 342 and elsewhere: What does the unit “m/m%” denote? Please use an SI unit.

L. 473, 532: spp. should not be in italics.

[Note: HTML markup is below. Please do not edit.]

Reviewers' comments:

Reviewer's Responses to Questions

**Comments to the Author**

Reviewer #1: All comments have been addressed

Reviewer #2: All comments have been addressed

**********

2. Is the manuscript technically sound, and do the data support the conclusions?

Reviewer #1: Yes

Reviewer #2: Yes

**********

3. Has the statistical analysis been performed appropriately and rigorously?

Reviewer #1: Yes

Reviewer #2: Yes

**********

4. Have the authors made all data underlying the findings in their manuscript fully available?

The PLOS Data policy

Reviewer #1: Yes

Reviewer #2: Yes

**********

5. Is the manuscript presented in an intelligible fashion and written in standard English?

Reviewer #1: Yes

Reviewer #2: Yes

**********

Reviewer #1: (No Response)

Reviewer #2: Thank you for carefully addressing all comments. I have one minor addition though: currently figure 1 caption looks incomplete. Please, review and revise.

**********

what does this mean?). If published, this will include your full peer review and any attached files.

If you choose “no”, your identity will remain anonymous but your review may still be made public.

**Do you want your identity to be public for this peer review?**  For information about this choice, including consent withdrawal, please see our Privacy Policy

Reviewer #1: No

Reviewer #2: No

**********

[NOTE: If reviewer comments were submitted as an attachment file, they will be attached to this email and accessible via the submission site. Please log into your account, locate the manuscript record, and check for the action link "View Attachments". If this link does not appear, there are no attachment files.]

To ensure your figures meet our technical requirements, please review our figure guidelines: https://journals.plos.org/plosone/s/figures

You may also use PLOS’s free figure tool, NAAS, to help you prepare publication quality figures: https://journals.plos.org/plosone/s/figures#loc-tools-for-figure-preparation.

NAAS will assess whether your figures meet our technical requirements by comparing each figure against our figure specifications.

# Author comment: Author response to Decision Letter 2

**DOI:** 10.1371/journal.pone.0357403.r004

14 Aug 2026

Response (R) to the Editor:

Thank you very much for your thorough editing, which has helped improve the quality of the manuscript. Below, we provide our responses to your comments. The line numbers refer to the unmarked version of the revised manuscript.

L. 140: Please replace “dried in” to “dried at”.

R: Thank you for your comment. We have corrected this as suggested (L. 140).

L. 155-157: Under the International Code of Nomenclature for algae, fungi, and plants (https://www.iapt-taxon.org/nomen/main.php), plant scientific names conventionally include a standard author abbrevia-tion, but omit the year of publication. Authority should not be italics, and authority should only be in pa-rentheses in cases where the original name of the species has changed. Please check and correct.

R: Thank you for your comment and for the detailed instructions. We apologise for not having checked these rules carefully enough. We have now corrected all the plant scientific names accordingly (L. 155-156).

L. 321, 325, 334, 552: Scientific names: Authority and year should not be in italics and only in parentheses in cases where the original name of the species has changed. Please check and correct, see International Commission on Zoological Nomenclature (https://www.iczn.org/outreach/faqs/).

R: Thank you for your comment and for the detailed instructions. We apologise for not having checked these rules carefully enough. We have now corrected all the scientific names accordingly (L. 324, 328, 337, 554).

L. 342 and elsewhere: What does the unit “m/m%” denote? Please use an SI unit.

R: Thank you for your comment. We agree that m/m% is not an SI unit and have therefore converted these values to g/kg (L. 345–348). We have also corrected the units in the Supplementary Material. As a result of this conversion, the numerical values are ten times higher.

L. 473, 532: spp. should not be in italics.

R: Thank you for pointing this out. We have corrected the formatting of spp. accordingly (L. 475, 534).

Response to Reviewer 2:

Thank you for carefully addressing all comments. I have one minor addition though: currently figure 1 caption looks incomplete. Please, review and revise.

R: Thank you for your comment. We have revised and expanded the caption of Figure 1 to provide the additional information needed to clarify the figure (L. 182-187).

Attachment Submitted filename: Response_to_Editor.docx

# Editor report: Decision Letter 2

**DOI:** 10.1371/journal.pone.0357403.r005

**Authors:** Mette Vestergård

17 Aug 2026

Fine-scale recovery of mite communities following local extinctions on grasslands

PONE-D-26-22862R2

Dear Dr. Veronika,

We’re pleased to inform you that your manuscript has been judged scientifically suitable for publication and will be formally accepted for publication once it meets all outstanding technical requirements.

Within one week, you’ll receive an e-mail detailing the required amendments. When these have been addressed, you’ll receive a formal acceptance letter and your manuscript will be scheduled for publication.

An invoice will be generated when your article is formally accepted. Please note, if your institution has a publishing partnership with PLOS and your article meets the relevant criteria, all or part of your publication costs will be covered. Please make sure your user information is up-to-date by logging into Editorial Manager at Editorial Manager® and clicking the ‘Update My Information' link at the top of the page. For questions related to billing, please contact billing support.

If your institution or institutions have a press office, please notify them about your upcoming paper to help maximize its impact. If they’ll be preparing press materials, please inform our press team as soon as possible -- no later than 48 hours after receiving the formal acceptance. Your manuscript will remain under strict press embargo until 2 pm Eastern Time on the date of publication. For more information, please contact onepress@plos.org.

Kind regards,

Mette Vestergård, Ph.D.

Academic Editor

PLOS One

Additional Editor Comments (optional):

Reviewers' comments:

# Editor report: Acceptance letter

**DOI:** 10.1371/journal.pone.0357403.r006

**Authors:** Mette Vestergård

PONE-D-26-22862R2

PLOS One

Dear Dr. Gergócs-Winkler,

I'm pleased to inform you that your manuscript has been deemed suitable for publication in PLOS One. Congratulations! Your manuscript is now being handed over to our production team.

At this stage, our production department will prepare your paper for publication. This includes ensuring the following:

* All references, tables, and figures are properly cited

* All relevant supporting information is included in the manuscript submission,

* There are no issues that prevent the paper from being properly typeset

You will receive further instructions from the production team, including instructions on how to review your proof when it is ready. Please keep in mind that we are working through a large volume of accepted articles, so please give us a few days to review your paper and let you know the next and final steps.

Lastly, if your institution or institutions have a press office, please let them know about your upcoming paper now to help maximize its impact. If they'll be preparing press materials, please inform our press team within the next 48 hours. Your manuscript will remain under strict press embargo until 2 pm Eastern Time on the date of publication. For more information, please contact onepress@plos.org.

You will receive an invoice from PLOS for your publication fee after your manuscript has reached the completed accept phase. If you receive an email requesting payment before acceptance or for any other service, this may be a phishing scheme. Learn how to identify phishing emails and protect your accounts at https://explore.plos.org/phishing.

If we can help with anything else, please email us at customercare@plos.org.

Thank you for submitting your work to PLOS One and supporting open access.

Kind regards,

PLOS One Editorial Office Staff

on behalf of

Dr. Mette Vestergård

Academic Editor

PLOS One
